# Population infection estimation from wastewater surveillance for SARS-CoV-2 in Nagpur, India during the second pandemic wave

**DOI:** 10.1371/journal.pone.0303529

**Published:** 2024-05-29

**Authors:** Edward Acheampong, Aliabbas A. Husain, Hemanshi Dudani, Amit R. Nayak, Aditi Nag, Ekta Meena, Sandeep K. Shrivastava, Patrick McClure, Alexander W. Tarr, Colin Crooks, Ranjana Lade, Rachel L. Gomes, Andrew Singer, Saravana Kumar, Tarun Bhatnagar, Sudipti Arora, Rajpal Singh Kashyap, Tanya M. Monaghan

**Affiliations:** 1 Department of Statistics and Actuarial Science, University of Ghana, Legon, Accra, Ghana; 2 School of Mathematical Sciences, University of Nottingham, University Park, Nottingham, United Kingdom; 3 Food Water Waste Research Group, Faculty of Engineering, University of Nottingham, University Park, Nottingham, United Kingdom; 4 Research Centre, Dr G.M. Taori Central India Institute of Medical Sciences (CIIMS), Nagpur, Maharashtra, India; 5 Dr B.Lal Institute of Biotechnology, 6-E, Malviya Industrial Area, Malviya Nagar, Jaipur, India; 6 National Institute for Health Research Nottingham Biomedical Research Centre, Nottingham University Hospitals NHS Trust, Nottingham, United Kingdom; 7 Queen’s Medical Centre, School of Life Sciences, University of Nottingham, Nottingham, United Kingdom; 8 Wolfson Centre for Global Virus Research, University of Nottingham, Nottingham, United Kingdom; 9 Nottingham Digestive Diseases Centre, School of Medicine, University of Nottingham, Nottingham, United Kingdom; 10 Nagpur Municipal Corporation, Nagpur, India; 11 UK Centre for Ecology and Hydrology, Wallingford, United Kingdom; 12 ICMR-National Institute of Epidemiology, Chennai, Tamil Nadu, India; Universidade Lisboa, Instituto superior Técnico, PORTUGAL

## Abstract

Wastewater-based epidemiology (WBE) has emerged as an effective environmental surveillance tool for predicting severe acute respiratory syndrome coronavirus 2 (SARS-CoV-2) disease outbreaks in high-income countries (HICs) with centralized sewage infrastructure. However, few studies have applied WBE alongside epidemic disease modelling to estimate the prevalence of SARS-CoV-2 in low-resource settings. This study aimed to explore the feasibility of collecting untreated wastewater samples from rural and urban catchment areas of Nagpur district, to detect and quantify SARS-CoV-2 using real-time qPCR, to compare geographic differences in viral loads, and to integrate the wastewater data into a modified Susceptible-Exposed-Infectious-Confirmed Positives-Recovered (SEIPR) model. Of the 983 wastewater samples analyzed for SARS-CoV-2 RNA, we detected significantly higher sample positivity rates, 43.7% (95% confidence interval (CI) 40.1, 47.4) and 30.4% (95% CI 24.66, 36.66), and higher viral loads for the urban compared with rural samples, respectively. The Basic reproductive number, *R*_0_, positively correlated with population density and negatively correlated with humidity, a proxy for rainfall and dilution of waste in the sewers. The SEIPR model estimated the rate of unreported coronavirus disease 2019 (COVID-19) cases at the start of the wave as 13.97 [95% CI (10.17, 17.0)] times that of confirmed cases, representing a material difference in cases and healthcare resource burden. Wastewater surveillance might prove to be a more reliable way to prepare for surges in COVID-19 cases during future waves for authorities.

## Introduction

Wastewater-based epidemiology (WBE) has emerged as a valuable and cost-effective strategy for monitoring the prevalence of severe acute respiratory syndrome coronavirus 2 (SARS-CoV-2) within communities and predicting disease outbreaks [1, 2]. This approach capitalizes on the detection of SARS-CoV-2 RNA in wastewater samples and has been widely employed using samples obtained from wastewater treatment plants (WWTPs) in nations with centralized sewage networks [1, 2]. While initially applied in countries with centralized sewage networks, predominantly through wastewater treatment plant (WWTP) samples [3, 4], the applicability of WBE has transcended geographical constraints, encompassing a diverse range of sources such as river water, airport wastewater, hospital effluents, marketplaces, and municipal drains [5–8].

This research endeavours to explore the feasibility of a cross-sectional wastewater-based sampling strategy aimed at detecting and quantifying SARS-CoV-2 viral loads in untreated wastewater within the Nagpur district, located in Maharashtra, Central India. Notably, the sampling done for this study coincides with the second wave of the COVID-19 pandemic in India in 2021, marked by an unprecedented surge in transmission and heightened disease impact. However, the comprehensive integration of WBE into disease surveillance systems, particularly in low- and middle-income countries (LMICs), is limited due to inadequate centralized sanitation facilities [3, 4]. One of the major reasons for this underutilization of WBE in LMICs, despite its huge potential, is that in such countries centralized sanitation facilities are often lacking [7].

In the course of carrying out this research, though there are several related works [9–13], one seminal study in the field of WBE that this research utilised was conducted by McMahon *et al*. [9]. Their study investigates the use of wastewater samples to monitor community-level transmission of SARS-CoV-2, the virus responsible for COVID-19. The authors employ a Susceptible-Exposed-Infectious-Recovered (SEIR) model to estimate the number of infected individuals based on SARS-CoV-2 RNA concentrations detected in wastewater. Via their rigorous analysis, McMahon *et al*. [14] demonstrate the utility of the SEIR model in predicting infections by considering various parameters such as transmission rates and viral shedding dynamics. In addition, their work introduces a simplified equation that aids in estimating infections from wastewater data, enhancing the accessibility of the model’s application. The study’s use of Monte Carlo simulations further strengthens the accuracy of predictions, revealing a notable discrepancy between estimated infections and confirmed cases, thus highlighting the potential value of the SEIR model in informing public health strategies [14].

To this end, the integration of wastewater-based estimates complements traditional clinical testing and bolsters the accuracy of surveillance efforts, especially in resource-constrained settings where extensive clinical testing might be challenging [15]. Thus, by integrating data from wastewater samples with demographic information and clinical data, a model is proposed which generates robust estimates of the number of COVID-19 infections within a given population. Crucially, this approach provides a comprehensive perspective on viral transmission dynamics, assisting public health officials in understanding the disease’s impact on a broader scale. The imperative of this study is to develop wastewater-based surveillance systems in LMICs, particularly those with resource limitations and complex infrastructural challenges and underscores the necessity of adapting WBE to a broader global context [16, 17]. In these settings, the translation of wastewater surveillance data into effective public health tools requires the integration of mathematical models and simulations.

To address these challenges, the adaptation of mathematical models is crucial. The use of a modified version of SEIR modelling and Monte Carlo simulation (MC) in this study is motivated by the ability to effectively capture and analyze the transmission dynamics of infectious diseases, such as SARS-CoV-2. The SEIR model and MC simulation have established themselves as valuable tools in epidemiological research because of their ability to provide insights into the complex systems involved in infection transmission, population dynamics, and uncertainty analysis. The SEIR compartment model forms the foundation for understanding disease transmission dynamics [18–22]. The SEIR model categorizes individuals into different compartments based on their disease status, encompassing susceptible, exposed, infectious, and recovered individuals. This model enables the estimation of disease prevalence over time, aiding in the interpretation of wastewater surveillance data and its linkage to community infection dynamics. MC simulations, on the other hand, are a robust computational technique used to account for uncertainties and variations in parameters. By generating multiple simulations with randomly sampled inputs, MC simulations enable the exploration of a range of possible outcomes. This is particularly valuable in epidemiological studies where factors such as contact rates, transmission probabilities, and intervention effects can vary or are uncertain. MC simulations provide a way to quantify the uncertainty associated with model predictions, helping researchers understand the potential variability in their results [14, 23–27].

This research initiative represents a pioneering effort in the Indian context, harnessing the SEIPR model and MC simulations to illuminate the transmission patterns of SARS-CoV-2 through wastewater. By addressing critical knowledge gaps within LMICs and regions confronting infrastructural limitations, this study contributes not only to scientific advancement but also furnishes actionable insights for policy formulation and disease mitigation. Amidst the complex landscape of the COVID-19 pandemic, this endeavour augments the global repository of knowledge, empowering communities and authorities alike to respond effectively to this ongoing public health challenge.

In this study, we explored the feasibility of conducting a cross-sectional wastewater-based sampling study for the detection, determination, and comparison of SARS-CoV-2 viral loads from untreated wastewater in urban and rural areas of Nagpur district, Maharashtra, Central India. We selected our sampling period during the second wave of COVID-19 in India in 2021. We next developed a modified version of the SEIR compartment mathematical model that has been frequently used to model COVID-19 dynamics in different populations [18, 19, 22], herein termed the “SEIPR model” to predict the number of infected individuals within specific Nagpur district partitioned zones and the total urban population under study. After predicting the number of infected individuals, the estimates were used to perform Monte-Carlo simulations to model the variations in the concentration of SARS-CoV-2 RNA in wastewater over time. These modelled changes were then compared to the actual measurements recorded to evaluate the accuracy of our SEIPR model. The urban incident COVID-19 cases were also used to calculate the basic reproduction number *R*_0_ based on the SEIPR model. This data was correlated to air temperature, relative humidity (a loose proxy for rainfall as we did not have the precise precipitation data), and population density to enhance epidemiological understanding of environmental and human factors that may impact SARS-CoV-2 transmission dynamics in Central India. To the best of our understanding, this is the first Indian report that has employed the SEIPR model to measure the transmission patterns of SARS-CoV-2 through wastewater. This study could prove valuable for local authorities and government officials as it provides important insights to make well-informed policy decisions.

## Materials and methods

### Wastewater sampling, SARS-CoV-2 detection, and quantification

**Fig 1:**
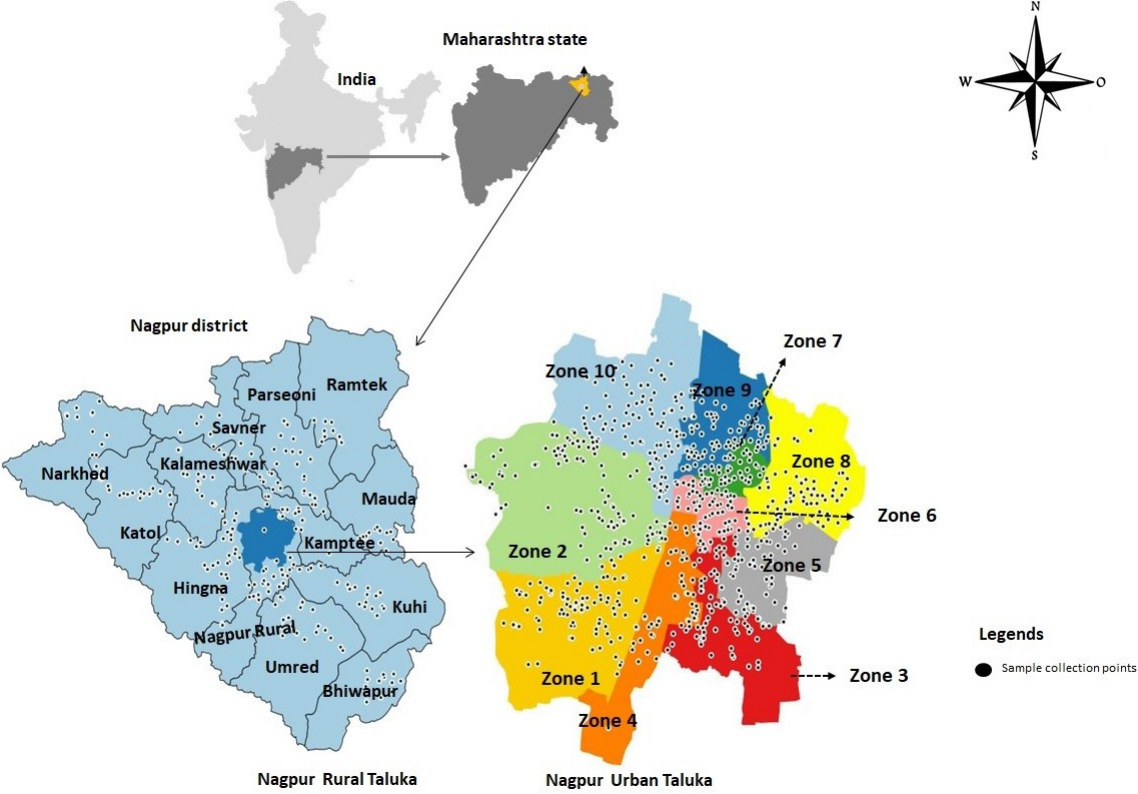
Map of Nagpur district (study area) showing sampling locations for wastewater study. Each dot represents a location of wastewater collection in Nagpur urban and rural talukas. The map was created using the ArcGIS 10.4 version from a GIS student. Source of map used “ESRI, Maxar, Earthstar, Geographics and the GIS user Community”.

Untreated (raw) wastewater samples were collected prospectively from the drainage systems in the Nagpur district of Maharashtra, India, during the second wave of the COVID-19 pandemic from January 31st to July 9th, 2021. Nagpur district is divided into 13 rural talukas and the Nagpur urban region, governed under Nagpur Municipal Corporation (NMC). The Nagpur urban region is further divided into ten municipality zones with each further divided into municipal wards. Individual grab samples were collected from sewers within each urban municipality zone as well as open drains/groundwater sources of rural talukas representing the complete Nagpur district, as illustrated in Fig 1 right panel (urban taluka) and left panel (rural talukas in relation to urban taluka). Each sample (1000 mL) was collected in sterile wide-mouth autoclaved plastic bottles sealed in plastic bags and transported under a cold chain at 4°C within 18–24 hours. All sampling was conducted during the morning hours between 07:30 to midday using appropriate COVID-19 precautions. Samples were transported to Dr B. Lal Institute of Biotechnology, Jaipur, for pre-processing, RNA extraction and SARS-CoV-2 detection by RT-qPCR, as previously described [28]. No specific permits were required for this study for field site access. We have only informed NMC regarding this study. Detailed sample processing methodology is presented in S1 Appendix.

### Data collection for COVID-19 cases and environmental characteristics

Demographics, and climatic factors including the presence of rainfall, air temperature, and relative humidity, along with GPS coordinates, were also recorded by field workers based at the Central Indian Institute of Medical Sciences (CIIMS) and assisted by the NMC. Daily laboratory-confirmed COVID-19 positive cases and deaths between 1st February and 30th July 2021 within the ten different municipality zones in urban Nagpur were obtained from the health department of the NMC.

### Epidemiological modelling and estimation of infected individuals

We based our study of the transmission of SARS-CoV-2 infections on a deterministic ordinary differential equation (ODE) disease model in which the individuals in an entire population can present in five mutually exclusive compartments according to their disease status and other measures. These compartments are susceptible, exposed, infectious, confirmed positive and recovered, abbreviated as the SEIPR model, which is a modification of the SEIR model and that described by Acheampong *et al*. [20], where additional compartments were given to reflect the Ghanaian environment [20]. We denote the proportion of susceptible individuals by *S*(*t*), the proportion of asymptomatic infected individuals by *E*(*t*), the proportion of symptomatic infectious individuals by *I*(*t*), the proportion of confirmed positive infectious individuals by *P*(*t*) and the proportion of recovered individuals by *R*(*t*). It must be noted here that individuals in the confirmed-positive class are carriers of the SARS-CoV-2 virus who have had clinical confirmation of this status. However, individuals in an infectious class show clear symptoms and have high infectivity but have not yet been clinically confirmed positive. Notably, as highlighted by Acheampong et al. [20], individuals classified within the infectious class *I*(*t*) represent an abstract concept that is often unmeasurable. This underscores the significance of introducing a compartment like the confirmed-positive class *P*(*t*), enabling comparison with the actual reported cases within the population. The SEIPR model was applied to study COVID-19 dynamics in ten zones within Nagpur’s urban area. Each zone operates independently. Disease transmission is driven by a force of infection (*λ*), determined by the effective contact rate per day (*β*_1_) and reductions in transmissibility for exposed (*β*_2_) and confirmed positive (*β*_3_) individuals. Disease-induced deaths are assumed to only occur within the infectious (*I*) and confirmed positive (*P*) compartments. The model describes how individuals transition between these compartments based on rates of entry and exit, such as exposure to infection (*λ*), testing (*ω*), recovery (*ρ*), and disease-induced death (*d*). Recovery of individuals (*R*) depends on recovery rates from the confirmed positive (*P*) and symptomatic infectious (*I*) compartments (*ρP*(*t*) and *ργP*(*t*), respectively). Additionally, no natural birth and death are considered, and their exclusion may be justified given the assumed short-term focus on COVID-19 dynamics and the neglect of population-level demographic changes, simplifying the model for this specific epidemiological context. These underlying assumptions guide the model’s representation of COVID-19 transmission and progression in the Nagpur urban area. The transmission dynamics of the SARS-CoV-2 infections are described by the five nonlinear systems of ODEs shown in Eq (1):

$$\begin{array}{c} \frac{\text{d}}{\text{dt}}{\text{X}}_{t}=\text{f}({\text{X}}_{t},\text{t},\theta ), \end{array}$$ (1)

with *X*_0_ = [*S*_0_, *E*_0_, *I*_0_, *P*_0_, *R*_0_]^*T*^ is initial number of individuals, t denotes time, *X*_*t*_ = [*S*, *E*, *I*, *P*, *R*]^*T*^ denotes the number of individuals in these compartments at time *t*, *T* denotes matrix transposition, denotes the parameter vector and *f*(⋅) denotes the nonlinear relationship describing the state variable (see S2 Appendix for detailed mathematical derivation of SEIPR model). The force of infection used in this model is λ = *β*_1_(*β*_2_*E*(*t*) + *β*_3_*P*(*t*) + *I*(*t*)), with *β*_1_ denoting the effective contact rate per day, and *β*_2_ and *β*_3_ respectively accounts for the reduction in disease transmissibility of exposed and confirmed positive individuals. A value of epidemiological importance in infectious disease modelling is the basic reproductive number, which in this study is referred to as the number of secondary SARS-CoV-2 infections generated by a single active SARS-CoV-2 infected individual during the entire infectious period [29]. It is given by the Eq (2):

$$\begin{array}{c} {\text{R}}_{0}=\beta_{1}{\text{S}}^{0}(\frac{\beta_{2}}{\epsilon }+\frac{\beta_{3}\left(1-\omega \right)}{i_{T}}+\frac{\delta \left(1-\omega \right)+i_{T}\omega }{i_{T}p_{T}}), \end{array}$$ (2)

where *i*_*T*_ = *δ* + *γρ* + *d* and *p*_*T*_ = *ρ* + *d*. The effective reproductive number (*R*_0_) is made up of contributions from secondary infections from the exposed class generated by asymptomatic individuals (first term), confirmed positive individuals’ class (second term), and the infected (symptomatic) class (third term). *S*^0^ is the proportion of the population that is initially susceptible. Other parameters in Eq (2) are defined as follows: *ϵ* denotes the incubation period, *σ* denotes the progression rate of susceptible individuals to the confirmed positive class via testing per day, *δ* denotes the progression rate of infectious individuals to the confirmed positive class via testing per day, *d* denotes the disease-induced death rate per day, *ω* denotes the fraction of exposed individuals that transient to confirmed positive class, *γ* denotes the fraction of infectious individual that transient to recovery class and *ρ* denotes the recovery rate of confirmed positive individuals per day. In this study, the nonlinear least squares scheme is used to estimate the parameters involved in the calculation of *R*_0_. The model fitting was first carried out for each zone to obtain zone-specific parameter estimates and secondly for all zones put together as a single unit. Further details about model derivation and parameter estimation can be found in the Supplementary (see S2 Appendix for a full description of model parameters and variables). For this study, the number of SARS-CoV-2 infected individuals within urban Nagpur was estimated using the modelling approach proposed by McMahon *et al*. [14], which combines our disease model (SEIPR) to the viral concentration estimations [14]. As already mentioned, there are ten zones within urban Nagpur and each zone is modelled independently. Based on McMahon *et al*. [14], using our SEIPR disease model, the number of newly detected infections on the *j*th day $I_{j}^{n}$ is modelled as a Poisson process with rate parameter *Nβ*_1_[*β*_2_*E*(*j*) + *β*_3_*P*(*j*) + *I*(*j*)], which is expressed as Eq (3):

$$I_{j}^{n}∼\text{Poisson}\{N\beta_{1}\left[\beta_{2}E\left(j\right)+\beta_{3}P\left(j\right)+I\left(j\right)\right]\},\;\text{for}\;j=1,2,\ldots ,J,$$ (3)

where *N* is the total number of individuals that reside in the zone of the drainage systems. The viral load being introduced into the drainage system at time *t* is

$$\begin{array}{c} V_{0}\left(t\right)=\sum_{j:j\leq t}\sum_{i=1}^{I_{j}^{n}}V_{ij}\left(t\right), \end{array}$$ (4)

where *V*_*ij*_(*t*) is the number of copies of SARS-CoV-2 RNA entering the drainage systems via faeces of the *i*th individual of out the $I_{j}^{n}$ who became infected on day *j* is modelled according to the Eq (5)

$$\begin{array}{c} V_{ij}\left(t\right)=\vartheta_{ij}\{10^{\frac{\phi_{ij}\left(t-j\right)}{5}}I\left(j<t\leq 5+j\right)+10^{\psi_{ij}^{\frac{\left(\phi_{ij}-\psi_{ij}\right)\left(t-5-j\right)}{5}}}I\left(t>5+j\right)\}, \end{array}$$ (5)

for $i=1,2,\ldots ,I_{j}^{n}$ (infected individuals) and *j* = 1, 2, …, *J* (days). In Eq (5), *ϑ*_*ij*_ denotes the *log*_10_ g of faeces per *i*th individual who gets infected on the *j*th day, modelled as a normal distribution with mean of 2.41 and standard deviation of 0.25 per data from lower-middle-income countries [15], *ϕ*_*ij*_ denotes the *log*_10_ maximum RNA copies per g being of faeces shed 5 days after being infected, modelled as a normal distribution with mean of 7.6 and standard deviation of 0.8 [14] and *ψ*_*ij*_ denotes the *log*_10_ RNA copies per g being of faeces shed 25 days after being infected, modelled as a normal distribution with mean of 3.5 and standard deviation of 0.4. To correlate the viral load being introduced into the drainage system to that being measured, McMahon *et al*. [14] proposed the Eq (6) called the downstream RNA copies measured, *V*(*t*, *τ*) to account for the time-dependent degradation in the drainage system,

$$\begin{array}{c} V\left(t,\tau \right)=V_{0}\left(t\right)(\frac{1}{2}{)}^{\tau /\tau^{*}}, \end{array}$$ (6)

where *τ* is the time elapsed between waste excretion and arrival at the drainage systems modelled as a uniform distribution from *τ* = 1h to *τ* = 1.5h, *V*_0_(*t*) is the viral load introduced into the drainage system modelled by Eq (4), τ* is the temperature-dependent half-life modelled according to Eq (7)

$$\begin{array}{c} \tau^{*}=\tau_{0}^{*}Q_{0}^{\left(T-T_{0}\right)/10^{\circ }C}, \end{array}$$ (7)

where *T* is the current temperature of the drainage system, modelled as a uniform distribution from *T* = 19°*C* to *T* = 31°*C*, $\tau_{0}^{*}$ is the half-life (h) at an ambient temperature of *T*_0_, modelled as a normal distribution with a means of 3 h and 30 h respectively, with standard deviations of 0.7 and 1.5, *Q*_0_ is the temperature-dependent rate of change, modelled as a normal distribution with a mean of 5.5 and standard deviation of 0.5. The choice of distributions and parameter ranges were informed by previous research as well as actual measurements or observations of SARS-CoV-2 in wastewater to inform their selection of parameter ranges for the Monte Carlo simulation. All the above information was used to simulate the viral load of infected individuals generated by our proposed disease model via 500 Monte Carlo simulations, since beyond this number of Monte-Carlo simulations, the value of the simulated RNA copies does not significantly change. Importantly, the number of Monte Carlo samples depends on various factors including the complexity of the model, which is the case here.

Finally, McMahon *et al*. [14] proposed a model for estimating the number of infected individuals in each day given the measured RNA copies quantified from samples collected from the drainage systems and is given by the Eq (8)

$$\begin{array}{c} J_{t}=\frac{Q\times V}{A\times B}, \end{array}$$ (8)

where *Q* denotes the average flow rate at the drainage system in L per day, *V* denotes the virus copies per L, *A* is the rate of faeces production per person in g per day with *A* = 2 × 128 for developing countries [15], and *B* denotes the maximum rate at which the virus is shed in RNA copies for g of faeces per day with *B* = 10^7.6^ × 128 [14]. In this study, *Q* was calculated as a point estimate using the product of the at-home population in the catchment of each zone, and the observed average per capita wastewater rate, which we assumed to be either 120 or 135 L/person/day (based on the Ministry of Housing and Urban affairs suggested benchmark for urban water supply).

### Statistical analyses

Due to the lack of COVID-19 incidence data for the rural areas in Nagpur, we explored catchment areas within urban Nagpur by zones to gain insight into the concentration of SARS-CoV-2 viral load in the collected wastewater samples. Based on the model parameter estimates, the distribution of the RNA copies per day existing in the drainage systems by zones was estimated, where we used the 2011 population census data as an estimate for each population zone. Of note, the use of the Monte Carlo simulation approach can help estimate uncertainties and account for variability in the data, which provides some indication of potential uncertainty and variability in prevalence estimates despite the limitations of using this census data, making the margin of error not a major problem. Data on continuous variables are presented as median with interquartile ranges (IQR). Categorical variables are shown as counts and percentages in parentheses. The normality of data was assessed using the Shapiro-Wilk test. Student’s t-test was used for comparing variables which were normally distributed. Mann-Whitney test was used when the normality assumption was violated. The Fisher’s exact test and Proportion tests were applied to compare categorical variables. All *p*− values and confidence intervals (CIs) are two-sided and a *p*–value of < 0.05 is considered statistically significant. All modelling studies were performed using MATLAB, 2022^*o*^ and Rstudio 2022.07.1+554 software on macOS Monterey Version 12.5.1 MacBook Pro (13-inch, M2, 2022).

### Ethical approvals

The study was approved by the Faculty of Medicine and Health Sciences Research Ethics Committee at the University of Nottingham (REC No. 131–1120), and the institutional ethics committees of the Central India Institute of Medical Sciences, Nagpuadjust width. Lal Institute of Biotechnology, Jaipur.

## Results

### SARS-CoV-2 detection in wastewater samples

A total of 983 wastewater samples were analysed, of which 743 (75.6%) were from the urban and 240 (24.4%) from the rural parts of Nagpur district. Overall, 43.7% (95% confidence interval 40.1, 47.4) of wastewater samples in the urban and 30.4% (95% confidence interval 24.66, 36.66), in rural areas tested positive for SARS-CoV-2 (*p* < 0.001); RT-PCR results revealed significantly higher SARS-CoV-2 viral copies per L in urban zones (*p* < 0.001). The median temperature of urban Nagpur was 29.0°C, (IQR: 25.75–31.00) and was significantly lower than that of the rural areas (31°C, (IQR: 29–33); *p* < 0.001). The median humidity was also significantly higher in urban (38%, IQR: 26–53) vs rural (32%, IQR: 22–50) Nagpur (*p* < 0.001) at the time of sampling (Table 1).

**Table 1:** Summary of climatic characteristics and RT-PCR results of wastewater samples collected within urban and rural Nagpur catchment.

Characteristics	Urban		Rural		Significance
	*N* = 743		*N* = 240		
Temperature (°C)	29.00	(25–75–31.00)	31.00	(29.00–33.00)	≤0.001
Humidity (%)	38.00	(26.00–53.50)	32.00	(22.00–50.00)	≤0.001
Seegene (RT-PCR)					
IC	26.00	(26.00–28.00)	25.00	(24.00–28.00)	≤0.05
E(Ct)	32.00	(31.00–33.00)	32.00	(32.00–36.00)	*n*.*s*
RdRp(Ct)	35.00	(34.00–36.00)	35.00	(34.00–36.00)	≤ 0.001
N(Ct)	33.00	(32.00–34.00)	32.00	(32.00–33.00)	≤ 0.001
Genome load (10^5^ copies per L)	1.40	(0.72–3.00)	1.17	(0.48–1.60)	≤ 0.001
RT-PCR Result^a^					
Positive	325	(43.74%)	73	(30.41%)	≤ 0.001
Negative	418	(56.26%)	167	(69.58%)	

Data are presented n (%) or median (IQR). NA = not applicable.

^a^: RT-PCR results for wastewater samples; ct: cycle threshold; n.s.: not significant

Of the 10 sampled urban catchment zones, two zones (7 and 9) yielded no SARS-CoV-2 RNA detection but did record the highest humidity levels (Table 2). Only 3 zones experienced rainfall; zones 1 and 8, where rainfall was recorded 1 day prior to sample collection, and zone 7, where sample collection took place during heavy rainfall. It is likely that these rainfall events would also contribute to diluting the sewage prior to sampling. Moreover, rainfall events would also contribute to more rapid and effective flushing out within the sewers. In zone 9, wastewater sampling followed the conclusion of the main COVID-19 infection wave, and therefore, the cases of COVID-19 at the time of sampling were expected to be very low, as illustrated in S1A–S1E Fig (S3 Appendix). The distributions of the continuous data and their normality plots by individual zones are shown in S3A–S3D Fig (S3 Appendix). The respective significance *p*– values shown on the plots are all less than 0.05, indicating the data is not normally distributed. S1 Table (S3 Appendix) summarises the demographic characteristics of the catchment zones where wastewater samples were collected. The demographic and environmental characteristics by zones are presented in S2 and S3 Tables (S3 Appendix).

**Table 2:** SARS-CoV-2 RT-PCR results detected per unit of time and detected viral load results of the wastewater samples with climatic and population census information for each Nagpur catchment zone.

Catchment	Population^a^	Temperature (°C)	Humidity (%)	RT-PCR Result^b^ (Positive)	Genome Copy (10^5^ Copies per L)
Zone 1	239171	24 (22–26)	52 (40–65)	24 (24.5)	1.135 (0.875–1.359)
Zone 2	159458	24 (22–26)	33 (25–39)	47 (39.5)	17.003 (3.463–298.375
Zone 3	232247	32 (30–34)	23 (18–34)	57 (87.7)	2.390 (0.776–3.753)
Zone 4	208426	30 (27.5–33.5)	21 (16.5–41)	39 (83.0)	0.883 (0.560–2.552)
Zone 5	243953	31 (30–32)	39 (27–45)	46 (73.0)	2.168 (1.533–2.864)
Zone 6	204438	31 (29–32)	36 (33–44.2)	36 (60.0)	2.509 (1.309–3.439)
Zone 7	187044	27 (26–29)	92 (88–94)	0 (0.0)	NA
Zone 8	346287	33 (30–34)	40 (28.5–52.5)	5 (6.7)	0.066 (0.036–0.124)
Zone 9	317321	29 (27–32)	83 (74–94)	0 (0.0)	NA
Zone 10	267320	27 (25–29)	27 (24–31)	71 (71.7)	0.705 (0.376–1.185)

Data are presented n (%) or median (IQR);

^a^: 2011 census data;

^b^: RT-PCR results for wastewater samples per unit of time for each zone,

NA: not available.

### Estimation of infected individuals

**Fig 2:**
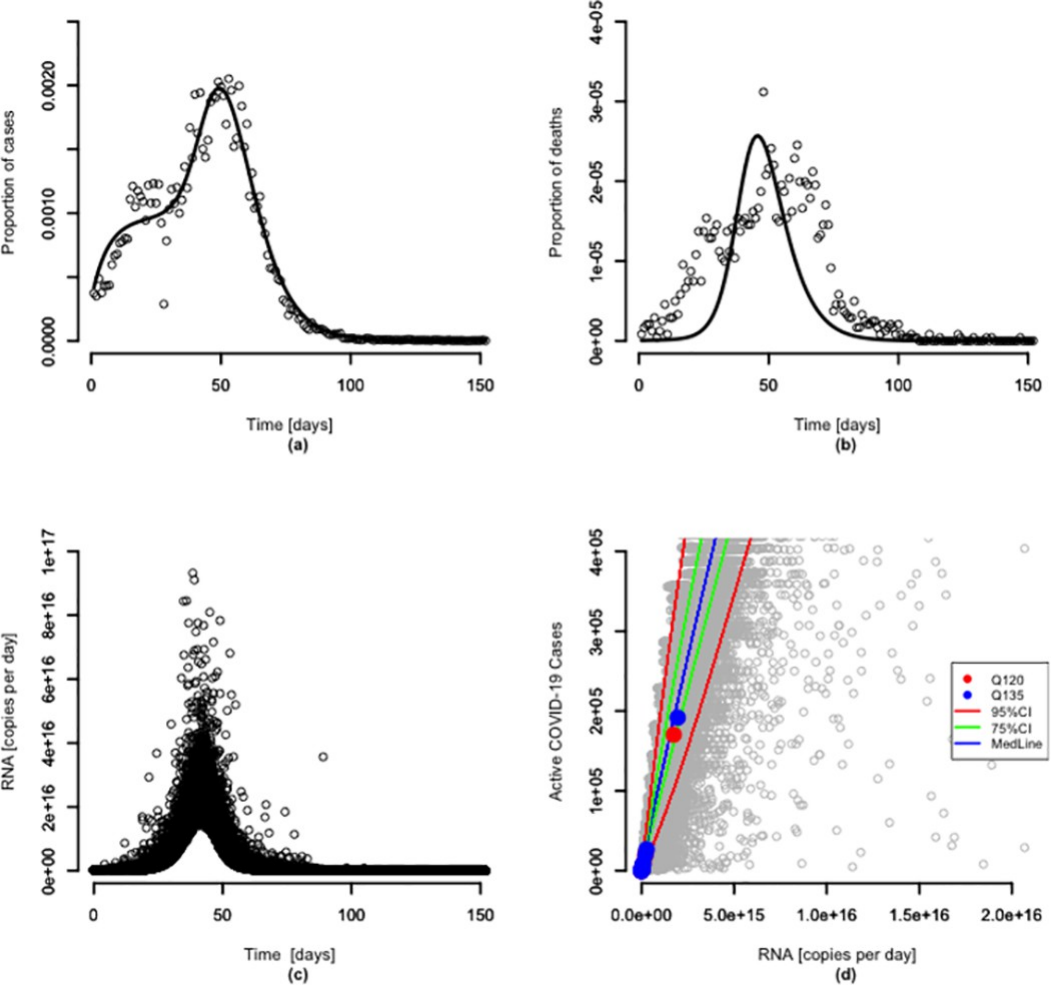
Considering a half-life of 30 h. Model fit to the proportion of the population. (a) (left) confirmed positive COVID-19 infections and (b) (right) confirmed deaths from COVID-19 infections for all zones as a single unit. (c) SEIPR model (1) prediction for the mass rate of SARS-CoV-2 RNA in wastewater over time via Monte-Carlo simulation represented by black points. (d) Zoomed-in plot of predicted number of active COVID-19 cases versus SARS-CoV-2 RNA mass rate with individual Monte-Carlo simulations represented by grey points, where 75% CI and 95% CI are denoted by the green and red solid lines, respectively. Coloured datapoints denote the measured RNA mass rates and estimated infectious individuals based on Eq (8) as presented in Table 4, respectively for an assumed average per capita wastewater rates of 120 L per person per day (red solid points) and 135 L per person per day (blue solid points) for all zones as a single unit.

We fitted our proposed SEIPR model to the reported confirmed COVID-19 positive cases and deaths in urban Nagpur via the nonlinear least squares method. Fig 2(a) and 2(b) shows the representative model fit for the SEIPR model to data for all 10 Nagpur catchment zones combined as a single unit for the period of March to July 2021.

Both plots show an increase in confirmed positive cases and deaths up to the first 50 days and then a decrease over the last 100 days. Thus, the SEIPR model predicts a decrease in the susceptible population as individuals become exposed, infected, confirmed positive, and then either recover or are confirmed dead. The remaining model fittings for the urban zones are presented in S5 Fig (S3 Appendix). The corresponding model parameter estimates for the respective catchment zones and *R*_0_ as calculated using clinical incident data only, are presented in Table 3. Each urban catchment zone exhibited different effective contact rates, *β*_1_, signifying different contact patterns. In addition, the basic reproduction number, *R*_0_ is different for each catchment zone with the highest *R*_0_ observed in zone 9 and lowest in zone 2. All the zones have an *R*_0_ greater than 1 except for zone 2. All the zones, when combined as a single unit, gave an *R*_0_ of 1.11. Linear regression analysis to investigate the variation in *R*_0_ and *β*_1_ between the zones revealed a statistically significant positive correlation between *R*_0_ and population density [*R*^2^ = 0.40, *p*–value = 0.05] whilst for effective contact rate (*β*_1_) and *R*_0_, there was a negative correlation with humidity [*R*^2^ = 0.49, *p*–value = 0.02]. No significant relationship was seen between temperature and *R*_0_ or *β*_1_ (S8 Fig in (S3 Appendix)).

**Table 3:** Model parameter estimates and basic reproduction number (*R*_0_) for each catchment zone of Nagpur district.

Catchment	Parameter										
	*β*_1_ (per day)	*β*_2_ (10^−4^)	*β*_3_ (10^−4^)	*ϵ* * (per day)	*σ* (10^−4^ per day)	*ω* (10^−4^ per day)	*δ* (10^−4^ per day)	*γ*	*ρ* (10^−4^ per day)	*d* (10^−4^ per day)	*R*_0_
Zone 1	0.91	0.42	143.79	0.2	2.48	19.16	21.81	1.00	1773.70	1.51	1.02
Zone 2	0.80	8.20	170.81	0.2	2.70	5.74	21.192	1.00	1627.00	1.58	0.98
Zone 3	0.92	0.01	8.22	0.2	2.64	10.13	25.811	1.00	1714.30	1.81	1.06
Zone 4	0.93	306.13	139.39	0.2	1.49	8.95	10.09	1.00	1344.40	1.03	1.52
Zone 5	0.99	7.12	1969.00	0.2	2.04	2.66	23.67	1.00	1557.30	1.88	1.26
Zone 6	0.76	1194.60	0.37	0.2	1.08	22.97	0.67	0.99	1333.90	0.56	1.59
Zone 7	0.75	0.02	11.05	0.2	0.522	2.70	3.51	1.00	1376.80	0.06	1.09
Zone 8	0.82	20.31	9990.80	0.2	0.69	1.66	8.46	0.98	1639.90	0.62	1.02
Zone 9	0.49	0.03	4998.80	0.2	0.88	27.16	0.01	0.59	1001.50	0.42	1.66
Zone 10	0.92	1.27	2.07	0.2	2.07	5.51	11.77	1.00	1583.00	0.87	1.15
All Zones	0.80	209.22	242.52	0.2	1.53	0.32	12.65	0.98	1570.60	0.98	1.11

*: fixed parameter estimate adapted from Zhang *et al*.,

**: Computation of *R*_0_ and all model parameters are based on clinical incidence data and not wastewater samples.

Note: *β*_1_ denotes the effective contact rate per day, *β*_2_ and *β*_3_ respectively account for the reduction in disease transmissibility of exposed and confirmed positive individuals. *ϵ* denotes the incubation period, *σ* denotes the progression rate of susceptible individuals to confirmed positive class via testing per day, *δ* denotes the progression rate of infectious individuals to confirmed positive class via testing per day, *d* denotes the disease-induced death rate per day, *ω* denotes the fraction of exposed individuals that transient to confirmed positive class, *γ* denotes the fraction of infectious individual that transient to recovery class and *ρ* denotes the recovery rate of confirmed positive individuals per day.

Taking all zones combined, Fig 2(c) depicts the distribution of the RNA copies per day, similar to the dynamics observed by McMahon *et al*. [14].

There is a positive correlation between the concentration of SARS-CoV-2 RNA in the wastewater and the number of confirmed positive individuals as well as recovering individuals and shedding rates. There was a different association between the measured viral RNA concentration and the confirmed positive cases during the earlier stages (January and February 2021) of the wastewater sampling, with high wastewater viral concentrations but low numbers of confirmed positive individuals. Therefore, zone 1 and zone 2 were not considered for the viral RNA load SEIPR modelling (see S2 Fig in the S3 Appendix). Fig 2(d) depicts a zoomed-in plot of the predicted number of active COVID-19 cases versus SARS-CoV-2 RNA mass rate with individual Monte-Carlo simulations represented by grey points. The measured RNA mass rates and estimated number of infectious individuals based on Eq (8) are denoted by the coloured datapoints and fall within the 95% CI denoted by the red solid lines. In this particular study, the sensitivity of the model regarding the viral half-life at an ambient temperature of the drainage is explored. It is observed that for a viral half-life of 3 h, the association between the mass rate of gene copies detected in wastewater and the confirmed positive cases is affected (see S7A Fig in the S3 Appendix). Data for all other catchment zones are given in the S6-S8 Figs (S3 Appendix)) except for zone 7 and zone 9, where wastewater samples from these zones tested negative (Table 2). Furthermore, all plots depicting the entire Monte-Carlo simulations of the predicted number of active COVID-19 cases versus SARS-CoV-2 RNA mass rate are presented in S9 and S10 Figs (S3 Appendix).

Table 4 presents the SARS-CoV-2 RNA wastewater concentrations in samples taken from all the catchment zones considered as a single unit between 1st March and 27th of May, 2021. Results of the other catchment zones are presented in the Supplementary. Each row corresponds to a specific date on which the wastewater samples were taken. The “RNA (copy per L)” column provides the concentration of SARS-CoV-2 genetic material in wastewater, providing insights into the prevalence of the virus in the population. The following columns, titled “Option 1” and “Option 2”, present two separate scenarios based on different wastewater rates per capita (120 L/person/day for Alternative 1). and 135 L/person/day for ‘Option 2). These scenarios are important for estimating the number of infected individuals using RNA concentrations as an indicator of viral activity. Calculated RNA levels are provided for each scenario, showing the rate of change in viral RNA levels per day. In addition, the “Estimated number of infected individuals” column quantifies the number of potential COVID-19 cases inferred from RNA levels, providing a way to assess community spread of the virus.

**Table 4:** SARS-CoV-2 RNA concentrations, estimated RNA rate and number of infected individuals from all the catchment zones as a single unit.

Date	RNA (10^5^ copies per L)*	Option 1		Option 2		Clinically observed number of Covid-19 positive cases
		RNA rate (10^14^ copies per day)^a^	Estimated number of infected individuals (10^14^)	RNA rate (10^14^ copies per day)^a^	Estimated number of infected individuals (10^14^)	
01/03/2021	6.10	1.76	17.28	1.98	19.40	777
02/03/2021	4.01	1.16	11.37	1.30	12.79	897
03/03/2021	3.50	1.01	9.91	1.14	11.15	845
04/03/2021	3.74	1.08	10.59	1.21	11.92	1172
08/03/2021	41.3 1	1.92	116.98	13.41	131.61	1049
09/03/2021	34.1	9.84	96.59	11.07	108.66	1433
10/03/2021	6.26	1.81	17.73	2.03	19.95	1604
05/04/2021	15.1	4.36	42.77	4.90	48.12	2652
06/04/2021	14.10	4.07	3.94	4.58	44.93	3283
07/04/2021	97.40	28.12	275.89	31.63	310.38	2881
08/04/2021	65.10	18.80	184.46	21.15	207.52	4016
13/04/2021	2.73	0.79	7.73	0.89	8.70	3613
15/04/2021	9.34	2.70	26.46	3.03	29.77	3779
19/04/2021	11.30	3.27	32.06	3.68	36.06	4878
20/04/2021	708.00	204.39	2005.44	229.93	2256.12	4787
21/04/2021	24.70	7.13	69.96	8.02	78.71	4619
23/04/2021	20.10	5.80	56.95	6.53	64.06	4936
24/04/2021	23.10	6.67	65.43	7.50	73.61	4720
27/04/2021	19.90	5.74	56.37	6.46	63.41	4803
28/04/2021	6.81	1.97	19.29	2.21	21.70	4422
29/04/2021	19.00	5.48	53.82	6.17	60.55	3649
30/04/2021	9.79	02.83	27.73	3.18	31.20	4085
03/05/2021	14.90	04.30	42.20	4.84	47.48	2498
04/05/2021	12.70	03.67	35.97	4.12	40.47	2534
06/05/2021	10.90	03.15	30.87	3.54	34.73	2255
07/05/2021	2.63	0.76	7.44	0.85	8.37	2016
25/05/2021	0.07	0.02	0.20	0.02	0.23	339
27/05/2021	0.18	0.05	0.52	0.06	0.58	216

Option 1 assumes an average per capita wastewater rate of 120 L/person/day; Option 2 assumes an average per capita wastewater rate of 135 L/person/day;

^a^: based on the numerator of Eq (8);

^b:^ based on Eq (8);

* aggregate SARS-CoV-2 RNA concentration if samples are taken from different locations measured on the day.

Direct comparison with clinically observed cases is presented in the column “Clinically observed COVID-19 positive cases”, showing actual confirmed positive cases reported by clinical diagnoses. This actual data is used as a benchmark to evaluate the validity of the estimates obtained through wastewater analysis. Side-by-side estimating infected individuals with observed clinical cases helps assess the reliability of using wastewater RNA concentrations as a predictor to monitor trends in COVID-19. Overall, this table highlights the importance of leveraging wastewater-based epidemiology to better understand viral prevalence. The ratio of unreported to reported cases under options 1 and 2 are respectively computed to be 12.42 (95% CI 9.04, 15.15) and 13.97 (95% CI (10.17, 17.0).

## Discussion

WBE has been used as a tool for surveillance of COVID-19 infections at the community-level and complements clinical-based surveillance and screening, which is limited by cost, turnaround time, and the bias associated with uncharacterized asymptomatic infections and their contribution to infection spread. WBE captures the totality of symptomatic, pre-symptomatic and asymptomatic carriers within a specific community [16, 17] This study is the first to successfully pilot and assess WBE as a methodology for the detection and quantification of SARS-CoV-2 viral RNA in community sewers in Nagpur district of Central India during the second wave of the pandemic in 2021. Whilst several epidemiological models have been described and compared for transmission of SARS-CoV-2 [2, 21, 30], this study employed a new SEIPR model, which adds the extra compartment of “confirmed positive” to estimate the number of infected individuals and was further used to estimate the mass rate of RNA in the wastewater. We observed a low number of clinical cases early in the COVID-19 wave that was out of proportion to the observed high SARS-CoV-2 concentration in the wastewater. If we use our modelling results from later in the study and apply them to this earlier period, it reveals that the clinical surveillance data underestimated the level of COVID-19 transmission in the Nagpur district. The model predicts the unreported number of cases under the per capita wastewater rates of 120L/person/day and 135L/person/day to be 12.42 (95% CI 9.04, 15.15) and 13.97 (95% CI (10.17, 17.0) times higher than the reported number of cases, respectively. Hence, SARS-CoV-2 RNA detected in community wastewaters may have come from pre-symptomatic, symptomatic, or asymptomatic cases who did not self-report to their local health monitoring unit due to fear of social stigma, isolation, or quarantine, or simply because they did not know they were infected [31, 32]. Under-reporting bias in the clinical incident data is also likely to have arisen due to the limitation of testing resources (analytical kits, personnel), coverage, and accessibility of testing sites [33]. We observed that SARS-CoV-2 seemed to be suppressed in samples collected from catchment zones recording higher relative humidity, a loose proxy for rainfall. This was further substantiated by observing a statistically significant negative correlation between *R*_0_ (effective reproductive number) and humidity, and *β*_1_ (effective contact rate per day) and humidity, but not temperature. These results partly agree with those reported elsewhere in which temperature and humidity were inversely correlated with daily new cases and deaths of COVID-19 with several studies reporting that SARS-CoV-2 is sensitive to high temperatures and humidity [34, 35]. It is likely that rainfall events prior to or during the sampling phase may have contributed to the lack of detection of SARS-CoV-2 RNA due to the dilutional effect. The substantial variation in parameter values across the ten geographic zones, as detailed in Table 3, is a consequence of the inherent complexity and diversity of real-world conditions being modelled. These variations are influenced by factors such as population density, healthcare infrastructure, interventions, and social behaviours specific to each zone. While these differences may appear significant, they are expected in epidemiological modelling and reflect the diverse nature of disease spread in different settings. Rather than indicating issues with the model, these variations underscore the need for tailored, context-specific modelling to capture the nuanced dynamics within each zone accurately. This diversity in parameters enhances the model’s ability to represent the unique characteristics of each zone. Moreover, the calculation of *R*_0_ considers the complex interplay of these parameters, and the model offers valuable insights into the dynamics of COVID-19 within a geographically diverse urban area like Nagpur. The variation in *R*_0_ estimates (0.98–1.66) between the different zones in Nagpur urban district may be due to additional factors such as variation in socio-behavioural habits (personal hygiene, wearing masks, handwashing, social distancing, vaccine uptake, social gatherings), sociodemographic, educational levels and dietary factors. Factors such as high levels of youth, income inequality, high population density and social media usage are associated with high *R*_0_ and may be important influences shaping zonal-wise variation in *R*_0_ in Nagpur as reported across countries [36]. Overall, these *R*_0_ estimates for the second wave of COVID-19 in India are consistent with a baseline *R*_0_ of 1.450 recorded for Maharashtra and 1.379 for India by Marimuthu *et al*. [37] but fall below earlier estimates calculated by Shil et al who reported *R*_0_ in the range of 2–3 during the initial wave of infection for the majority of Indian districts (March-June 2020) [38]. This depicts that the use of 2011 population census data as a proxy for the modelling process in this study was not out of place as the estimated *R*_0_ in this study is consistent with what other studies have found. This feasibility study identified a unique set of challenges in the implementation of WBE in Central India which mirrors those observed in other LMIC settings such as Bangladesh [4]. These include establishing a sampling plan and schedule that is representative of the different urban and rural catchment populations, underdeveloped sewage systems in rural areas necessitating onsite sanitation epidemiology/sampling; development and validation of standardized protocols for lab analysis; complex collaborative efforts from government agencies, public health units and academia and resource limitations (e.g., autosamplers not suitable for large rapid monitoring where passive sampling techniques are more easily implemented) [39]. Supply chain issues for essential goods such as PPE and PCR diagnostic reagents, and logistical constraints such as inaccessibility and poor transport systems made it difficult to reach rural communities in remote areas. In recognition of these challenges, we acknowledge several study limitations. Although we did assess and compare the abundance of SARS-CoV-2 viral concentration in untreated wastewater samples between urban and rural areas, in line with other wastewater research studies in India [40, 41] most of our sampling sites were from urban zones of Nagpur, introducing sampling bias. Due to the lack of COVID-19 clinical incident data for the rural areas sampled, we were not able to apply our SEIPR model to model infectious burden in rural Nagpur. We also had to base our model assumptions on historical rather than current census data which is not available from Nagpur district. Due to the limitation of resources and skilled personnel, we were not able to undertake 24-hour composite and longitudinal sampling which we recognize would have made our data more representative, to assess the impact of seasonality or to obtain detailed information on spatiotemporal trends. Moreover, we were unable to record physiochemical, hydrologic, and anthropogenic parameters of the wastewater samples which would have affected RNA concentrations, and consequently, SARS-CoV-2 RNA detection [31]. Although we did not collect daily rainfall measurements and instead used relative humidity as a proxy for rainfall, the majority of the sampling period was conducted during periods of no rain. We acknowledge the use of air temperature as a surrogate for wastewater temperature in the absence of direct wastewater temperature data, particularly in open drainage systems. While this substitution is a common practice in environmental modelling due to data limitations, it’s essential to recognize its potential limitations and the possible impact on the results. Wastewater temperature can be influenced by various factors beyond just air temperature in open drainage systems, such as ground temperature, flow rates, and interactions with other environmental factors. This assumption may introduce some level of uncertainty into the model, and future studies should aim to collect specific wastewater temperature data to improve the accuracy of the modelling. However, given the data constraints, the use of air temperature can provide a reasonable estimation of wastewater temperature and is a common approach in the field. We recognize that with any modelling efforts, it will be important to explore the sensitivity of the model to different assumptions in future research. Future studies should also adopt the use of rapid in-field testing of SARS-CoV-2 or any pathogenic target as opposed to bringing samples back to a central lab with appropriately trained personnel. This technology is already in proof-of-concept stages and could be easily operationalized ahead of future outbreaks or pandemics.

## Conclusion

We have established a quantitative framework to estimate COVID-19 prevalence and predict SARS-CoV-2 transmission through integrating wastewater-based surveillance data into a SEIPR model. The constructed model may be used to provide accurate and robust estimates of future waves of the COVID-19 pandemic and could usefully be applied to study other infectious diseases or expanded to consider reinfected populations. Our findings showcase the translational value of utilizing WBE to study the health of a population for epidemiological inference and in informing public health actions, particularly where comprehensive individual testing is severely constrained by a shortage of resources and logistical challenges. However, to realize the true value of this tool in India and other LMICs, it will be important for governmental and other funding agencies to invest heavily in building laboratory capacity and sample collection teams. Such efforts should also help re-emphasize the criticality of clean water, sanitation, and waste management as potential control points in the fight against COVID-19 and future pandemics.

## Supporting information

S1 Appendix Pre-processing of wastewater samples, nucleic acid extraction and SARS-CoV-2 qualitative and quantitative detection. (PDF)

S2 Appendix Epidemiological model formulation and fitting. (PDF)

S3 Appendix Figures and tables. (PDF)
