# Supplementary material for: Population infection estimation from wastewater surveillance for SARS-CoV-2 in Nagpur, India during the second pandemic wave
Source: PLoS One. 2024 May 29;19(5):e0303529. doi: 10.1371/journal.pone.0303529 (PMC11135679; doi:10.1371/journal.pone.0303529)
Supplement: S1 Appendix — (PDF) [file pone.0303529.s001.pdf]

Population infection estimation from wastewater surveillance for SARS-CoV-2 in Nagpur, India during the second pandemic wave

Edward Acheampong*, Aliabbas A Husain, Hemanshi Dudani, Amit R Nayak, Aditi Nag, Ekta Meena, Sandeep K Shrivastava, Patrick McClure, Alexander W Tarr, Colin Crooks, Ranjana Lade, Rachel L Gomes, Andrew Singer, Saravana Kumar, Tarun Bhatnagar, Sudipti Arora*, Rajpal Singh Kashyap* Tanya M Monaghan*

* Main correspondence:
Tanya M. Monaghan (tanya.monaghan@nottingham.ac.uk)

S1 Appendix. Pre-processing of wastewater samples, nucleic acid extraction and SARS-CoV-2 qualitative and quantitative detection.

Pre-processing of wastewater samples

All grab wastewater samples were collected in Nagpur district and sent to Dr. B. Lal Institute of Biotechnology, Jaipur. The received samples were immediately stored at 4°C and further processed within 24 hours of receiving. The pre-processing method involved surface sterilization by providing UV light to the receptacles for 30 minutes, which were then placed in a sonicator water bath at 60-70°C and incubated for 90 minutes for heat inactivation of the virus. After the inactivation process, the samples were brought to room temperature. The grit particles were removed by crude filtration using Whatmann filter paper and then fine filtered through $0.45\mu m$ membranes using a vacuum filter assembly. The filtrate of each respective sample was transferred to a fresh 50 mL falcon containing 0.9 g sodium chloride (NaCl) and 4 g polyethylene glycol (PEG). The contents were dissolved by gentle manual mixing. The samples containing PEG and NaCl were then centrifuged at 4°C for 30 minutes at 5750 g. The pellet obtained was further re-suspended in 1x sterile phosphate buffer saline (PBS) for automated extraction [1, 2].

Nucleic acid extraction

The RNA genome of virus was extracted from the pre-processed wastewater samples using the automated KingFisher™ Flex machine using the MagMAX Viral/Pathogen Nucleic Acid Isolation Kit (Applied Biosystems, Waltham, MA USA), as per the kit's instructions, as described in Arora *et al.* [3]. The protocol involves three wash steps for the extraction of the RNA. Samples were vortexed for 10 seconds and then mixed with the extraction buffer consisting of binding solution, binding beads and Proteinase K (referred to as the extraction master mix). The samples were next vortexed for 30 seconds and then processed using the automated system. This was followed by three washing steps using wash Plate 1 consisting of wash buffer, and wash plate 2 and 3, each consisting of varying amounts of 80% PCR grade ethanol. The RNA was eluted out with the process taking 24 minutes each time. The eluted RNA aliquots were kept

in the 96-well deep plates and were then sealed and stored at -20 °C until further use.

SARS-CoV-2 qualitative and quantitative detection

The qualitative and quantitative presence of SARS-CoV-2 RNA was detected in the total RNA extracted from the wastewater samples using CFX 96 Thermal Cycler (Bio-Rad) RT-PCR machine using 2 separate commercially available kits as described in Arora *et al.* [3].

The Allplex™ 2019- nCoV Assay RT-PCR (kit 1) was used for the qualitative detection of SARS-CoV-2 and consisted of 2019-nCoV MOM (prepared master mix), 5x Real-time One-step Buffer, Real-time One-step Enzyme. Since it was not possible at the time of the study to import a positive control and under the urgent circumstances, only MS2 phage was used as exogenous Internal process Control (IC). The kit 1 targeted *E* gene, *N* gene and *RdRp* gene with FAM and HEX as internal controls and were read on Cal Red 610 and Quasar 670 fluorophore channels, respectively. The PCR reaction was set up by mixing 11 μL of the isolated RNA with 14 μL of the RT-PCR master mix. The reaction protocol consisted of 1 cycle at 50°C for 20 minutes, 1 cycle at 95°C for 15 minutes followed by 45 cycles of denaturation at 94°C for 15 seconds and combined annealing and extension for 30 seconds at 58°C followed by plate read and detection. The PCR run was analyzed with Bio-Rad CFX Manager software version 3.1 (Bio Rad Laboratories). As per the manufacturer's instructions, the detection of a minimum of any two genes (out of three) in a sample was considered positive based on Ct values [3]. Positive samples through these criteria were further quantified.

To further quantify the presence of SARS-CoV-2 viral genome in the wastewater samples, InnoDetect One Step COVID-19 (Kit 2) was used wherein plasmid DNA consisting of the *N* gene was used to prepare a standard curve (a range of 10pg/μL to 0.01pg/μL) as per the protocol in the manufacturer's instructions [4]. These standard curves were then used for the quantification of the *N* gene in the samples. RNase free water was used to make a main stock of concentration of 40ng/μL. Kit 2 consists of a master mix, primer probe (*N* gene, ORF1ab & RNaseP) and uses three fluorophore channels (HEX/VIC, FAM & ROX/Texas Red, respectively). Viral RNA of SARS-CoV-2 was used as a positive control and DNase RNase free water as a negative control provided with the kit. The reaction cycle consists of a reverse transcription step at 42°C for 15 minutes 1 cycle, cDNA initial denaturation at 95°C for 3 minutes 1 cycle, denaturation at 95°C for 15 seconds and combined annealing and extension at 60°C for 40 seconds, followed by plate reading and detection.

Quality assurance metrics for justifying real-time PCR quantitative data were maintained through ensuring strong linear relationships by plotting the Ct (cycle threshold) values against the logarithm of the starting quantity of the standard samples, calculation of the slope and y-intercept, parameters which indicate the efficiency and baseline Ct value of the PCR assay, respectively and calculation of the R^2 value to evaluate the goodness of fit of the standard curve. The standard curve analysis indicated the amplification efficiency of the PCR assay for FAM and HEX internal controls as 103.1% and 113%, respectively. Considering that RNA extracted from wastewater might have inhibitors which might affect the detection of one or more target genes, positive RNA samples extracted from nasopharyngeal swabs were used in each run. Similar, clinical RNA samples which were confirmed negative as per ICMR guidelines were used in every run to validate negative samples. The limits of detection of the PCR assay were 500 copies/mL. The real-time PCR instrument was also regularly validated and checked in accordance with the manufacturers guidelines to ensure accurate temperature control, fluorescence detection, and signal normalization.

References

1. Arora S, Nag A, Sethi J, Rajvanshi J, Saxena S, Shrivastava SK, Gupta AB. Sewage surveillance for the presence of SARS-CoV-2 genome as a useful wastewater based epidemiology (WBE) tracking tool in India. Water Science and Technology. 2020; Dec 15;82(12):2823-36.

2. Arora S, Nag A, Rajpal A, Tyagi VK, Tiwari SB, Sethi J, Sutaria D, Rajvanshi J, Saxena S, Shrivastava SK, & Srivastava V. Imprints of lockdown and treatment processes on the wastewater surveillance of SARS-CoV-2: A curious case of fourteen plants in Northern India. Water. 2021; 13(16), 2265.

3. Arora S, Nag A, Kalra A, et al. Successful application of wastewater-based epidemiology in prediction and monitoring of the second wave of COVID-19 with fragmented sewerage systems – a case study of Jaipur (India). Environ. Monit. Assess. 2022; 194(5): 342.

4. Nag A, Arora S, Sinha V, et al. Monitoring of SARS-CoV-2 Variants by Wastewater-Based Surveillance as a Sustainable and Pragmatic Approach – A Case Study of Jaipur (India). Water. 2022; 14: 297.
