# Supplementary material for: Population infection estimation from wastewater surveillance for SARS-CoV-2 in Nagpur, India during the second pandemic wave
Source: PLoS One. 2024 May 29;19(5):e0303529. doi: 10.1371/journal.pone.0303529 (PMC11135679; doi:10.1371/journal.pone.0303529)
Supplement: S2 Appendix — (PDF) [file pone.0303529.s002.pdf]

Population infection estimation from wastewater surveillance for SARS-CoV-2 in Nagpur, India during the second pandemic wave

Edward Acheampong*, Aliabbas A Husain, Hemanshi Dudani, Amit R Nayak, Aditi Nag, Ekta Meena, Sandeep K Shrivastava, Patrick McClure, Alexander W Tarr, Colin Crooks, Ranjana Lade, Rachel L Gomes, Andrew Singer, Saravana Kumar, Tarun Bhatnagar, Sudipti Arora*, Rajpal Singh Kashyap* Tanya M Monaghan*

* Main correspondence:
Tanya M. Monaghan (tanya.monaghan@nottingham.ac.uk)

S2 Appendix. Epidemiological model formulation and estimation.

Epidemiological model formulation.

This section presents a brief description of the proposed model used to capture the dynamics of infectious COVID-19 individuals within ten zones in Nagpur urban area of India. The proposed model is a modification of a model used in Acheampong *et al.* [1]. The total population within each zone at any time t, denoted by $N(t)$ is respectively partitioned into five mutually exclusive compartments depending on the status of the disease. These compartments are the proportion of susceptible individuals $S(t)$, proportion of non-symptomatic infected individuals $E(t)$, proportion of symptomatic infectious individuals $I(t)$, proportion of confirmed positive infectious individuals, $P(t)$ and proportion of recovered individuals, $R(t)$. That is, susceptible, exposed, infectious, confirmed positive and recovered model, which is abbreviated as SEIPR. Each zone is modelled independently. The proportion of individuals in the respective compartments: $E(t)$, $I(t)$, and $P(t)$ can transmit the infection. Thereby, the force of infection is

$$\lambda = \beta_1(\beta_2 E(t) + \beta_3 P(t) + I(t)), \tag{1}$$

where β_1 is the effective contact rate per day, β_2 and β_3 respectively account for the reduction in disease transmissibility of exposed and confirmed positive individuals. It is further assumed that disease-induced death only happens to individuals in $I(t)$ and $P(t)$, compartments. Thus, the following five nonlinear systems of differential equations are used to model the transmission dynamics of the COVID-19 infections within each zone:

$$\frac{d}{dt}S(t) = -(\lambda + \sigma)S(t). \tag{2}$$

It is assumed that no new susceptible, $S(t)$ individuals enter this compartment. The proportion of individuals in the susceptible, $S(t)$ compartment may decrease either when individuals get tested positive for the infection and enter the confirmed positive compartment at the rate $\sigma S(t)$ or when individuals acquire the infection and enter the asymptomatic infected compartment at the rate $\lambda S(t)$.

$$\frac{d}{dt}E(t) = \lambda S(t) - \epsilon E(t). \tag{3}$$

The proportion of individuals in the exposed, $E(t)$ compartment increases at the same rate susceptible individuals get infected. The proportion of exposed individuals deceased at the rate $\epsilon E(t)$, of which the fraction of the individuals get tested positive for the infection and enters the confirmed positive compartment at the rate $\omega\epsilon E(t)$ and fraction of individuals that becomes infectious and symptomatic enter the symptomatic infectious compartment at the rate $(1-\omega)\epsilon E(t)$.

$$\frac{d}{dt}I(t) = (1-\omega)\epsilon E(t) - (\delta + \gamma\rho + d)I(t). \tag{4}$$

Infected individuals become symptomatic at the rate $(1-\omega)\epsilon E(t)$. Symptomatic infectious individuals may leave the compartment via recovery at the rate $\gamma\rho I(t)$ or via testing positive for the infection and enter the confirmed positive compartment at the rate $\delta I(t)$. It also decreases due to infection-induced death rate $dI(t)$.

$$\frac{d}{dt}P(t) = \sigma S(t) + \omega\epsilon E(t) + \delta I(t) - (\rho + d)P(t). \tag{5}$$

Newly confirmed positive infections enter the compartment at the same rate they get tested positive from susceptible, exposed, and infectious compartments. The proportion of confirmed positive individuals may leave the compartment via diseased-induced death rate of $dP(t)$ or via recovery after receiving treatment at the rate of $\rho P(t)$.

$$\frac{d}{dt}R(t) = \rho(P(t) + \gamma I(t)). \tag{6}$$

The proportion of recovered individuals increases due to recovery at the rates $\rho P(t)$ and $\rho\gamma I(t)$, respectively from confirmed positive and symptomatic infectious individuals. The SEIPR flow diagram of COVID-19 disease is depicted in Fig 1.

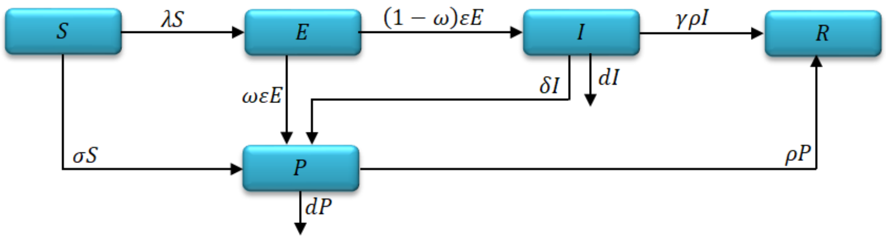

Fig S1. Flowchart for the model of COVID-19 transmission involving five compartments. See Table S1 for explanations of the parameters and variables used in the model.

The basic reproductive number

The basic reproduction number in this study is referred to as the number of secondary COVID-19 infections generated by a single active COVID-19 infected individual during the entire infectious period [1]. Mathematically, the basic reproduction number R_0 is the dominant eigenvalue of the next generation matrix [2, 3]. In this study, the method formulation in Van den Driessche and Watmough [2] is applied to obtain an expression of R_0 for the proposed SEIPR model. Let $\mathbf{x} = (E, I, P)^T$, then the system of differential Eqs (1-6) can be written in the form

$$\frac{d\mathbf{x}}{dt} = \mathcal{F}(\mathbf{x}) - \mathcal{V}(\mathbf{x}). \tag{7}$$

Table S1. Description of model parameters and state variables.

variable	Description
S	Proportion of susceptible population
E	Proportion of non-symptomatic infected (exposed) population
I	Proportion of symptomatic infectious population
P	Proportion of confirmed positive population
R	Proportion of recovery population
**Parameter**	**Description**
λ	Force of infection
β_1	Effective contact rate
β_2	Accounts for reduction in disease transmissibility of exposed individual
β_3	Accounts for reduction in disease transmissibility of confirmed positive individual
ϵ	Incubation period
σ	Progression rate of susceptible individual to confirmed positive class via testing per day
δ	Progression rate of infectious individual to confirmed positive class via testing per day
d	Disease-induced death rate per day
ω	Fraction of exposed individuals that transient to confirmed positive class
γ	Fraction of infectious individuals that transient to recovery class
ρ	Recovery rate of confirmed positive individuals per day

where

$$\mathcal{F}(\mathbf{x}) = \begin{pmatrix} \beta_1(\beta_2 E + \beta_3 P + I)S \\ 0 \\ 0 \end{pmatrix}, \quad \mathcal{V}(\mathbf{x}) = \begin{pmatrix} \epsilon E \\ -(1-\omega)\epsilon E + i_T I \\ -\sigma S - \omega\epsilon E - \delta I + p_T P \end{pmatrix}. \tag{8}$$

The corresponding Jacobian of $\mathcal{F}(\mathbf{x})$ and $\mathcal{V}(\mathbf{x})$ evaluated at the disease-free equilibrium E_0 are, respectively given by

$$F) = \begin{pmatrix} \beta_1\beta_2 & \beta_1\beta_3 & \beta_1 \\ 0 & 0 & 0 \\ 0 & 0 & 0 \end{pmatrix}, \quad V = \begin{pmatrix} \epsilon & 0 & \\ \epsilon(\omega-1) & i_T & 0 \\ -\omega\epsilon - \delta + p_T & & \end{pmatrix}, \tag{9}$$

where $i_T = \delta + \gamma\rho + d$ and $p_T = \rho + d$. Hence, the basic reproduction number, R_0 is given by the dominant eigenvalue of FV^{-1}, which is

$$\mathrm{R}_0 = \beta_1 \mathrm{S}^0 \left(\frac{\beta_2}{\epsilon} + \frac{\beta_3(1-\omega)}{i_T} + \frac{\delta(1-\omega) + i_T\omega}{i_T p_T} \right), \tag{10}$$

$$= \mathrm{R}_E + \mathrm{R}_P + \mathrm{R}_I, \tag{11}$$

where the effective reproduction number, R_0 is made up of contributions from secondary infections from the exposed class $E\,(R_E)$ generated by asymptomatic individuals; confirmed positive individuals — class $P\,(R_P)$; and the infected (symptomatic) class I (R_I). S^0 is the proportion of the population that is initially susceptible. Eq (10) implies the classes $(E, I,$ and $P)$ are the main drivers of the infections and as such, that intervention strategies of COVID-19 infections should target those in these classes. According to Theorem 3.2 of Van den Driessche and Watmough [2], the disease-free steady state E^0 is locally asymptotically stable if $R_0 < 1$ and unstable if $R_0 > 1$. Here, the disease-free steady state is given by

$$E^0 = (S^0, 0, 0).$$

Epidemiological model fitting and Non-linear least squares estimation

In this section, the least squares estimation technique used for model parameter estimation is briefly described. Note that the SEIPR model given by Eqs (2-6) can be generally expressed in the form

$$\frac{d\Upsilon}{dt} = F(t, \Upsilon, \theta), \qquad \Upsilon(t_0) = \Upsilon_0, \tag{12}$$

where the non-linear function F depends on time t, the vectors of dependent or state variables Υ and unknown model parameters θ to be estimated. The goal of the least squares estimation is to obtain the best values of the model parameters by minimizing the error between the reported data points $\Upsilon_(t_i)^d ata$ and the solution $\Upsilon_(t_i)^s im$ of the model given by Eqs (2-6) associated with the model parameters θ. The objective function used in the minimization task is given by

$$C(\theta) = \sum_{i=1}^{n} \left(\Upsilon_(t_i)^d ata - \Upsilon_(t_i)^s im \right)^2, \tag{13}$$

where n is the available reported data points. Thus, the non-linear least squares estimate of the model parameters, θ is

$$\hat{\theta} = \arg\min_{\theta} C(\theta). \tag{14}$$

References

1. Acheampong E, Okyere E, Iddi S, et al. Mathematical modelling of earlier stages of COVID-19 transmission dynamics in Ghana. Results Phys. 2022; 34: 105193.

2. van den Driessche P, Watmough J. Reproduction numbers and sub-threshold endemic equilibria for compartmental models of disease transmission. Math. Biosci. 2002; 180: 29–48.

3. Diekmann O, Heesterbeek JA, & Roberts MG. The construction of next-generation matrices for compartmental epidemic models. J R Soc Interface, 7(47), 873-885. https://doi.org/10.1098/rsif.2009.0386
