# Supplementary material for: Population infection estimation from wastewater surveillance for SARS-CoV-2 in Nagpur, India during the second pandemic wave
Source: PLoS One. 2024 May 29;19(5):e0303529. doi: 10.1371/journal.pone.0303529 (PMC11135679; doi:10.1371/journal.pone.0303529)
Supplement: S3 Appendix — (PDF) [file pone.0303529.s003.pdf]

Population infection estimation from wastewater surveillance for SARS-CoV-2 in Nagpur, India during the second pandemic wave

Edward Acheampong*, Aliabbas A Husain, Hemanshi Dudani, Amit R Nayak, Aditi Nag, Ekta Meena, Sandeep K Shrivastava, Patrick McClure, Alexander W Tarr, Colin Crooks, Ranjana Lade, Rachel L Gomes, Andrew Singer, Saravana Kumar, Tarun Bhatnagar, Sudipti Arora*, Rajpal Singh Kashyap* Tanya M Monaghan*

* Main correspondence:
Tanya M. Monaghan (tanya.monaghan@nottingham.ac.uk)

S3 Appendix. Figures and Tables.

Figure S1A: Bar-plot of the number of wastewater samples collected within the 10 catchment zones at specific dates (30/01 — 16/02).

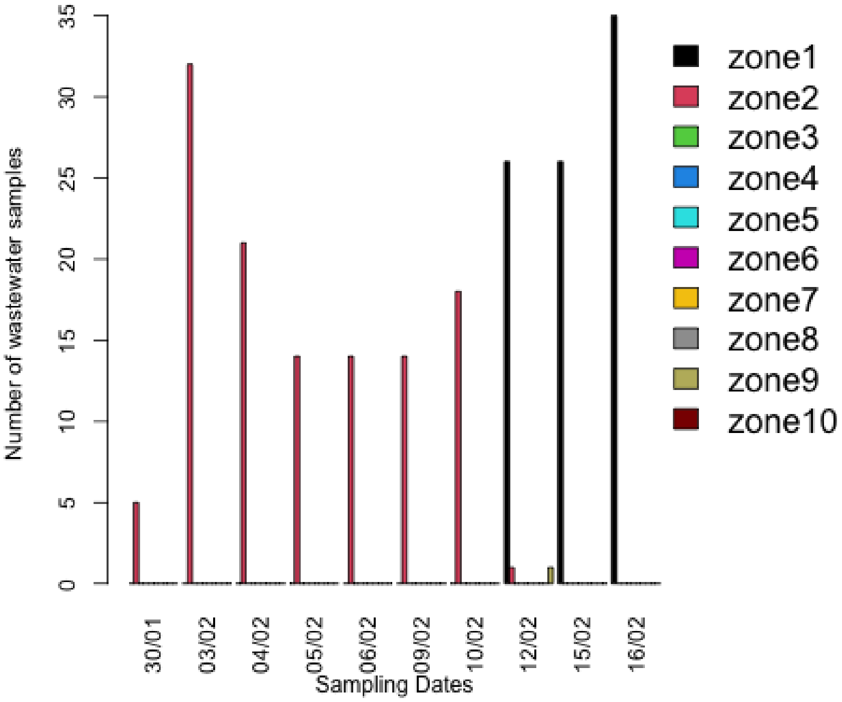

Figure S1B: Bar-plot of the number of wastewater samples collected within the 10 catchment zones at specific dates (17/02 — 09/03).

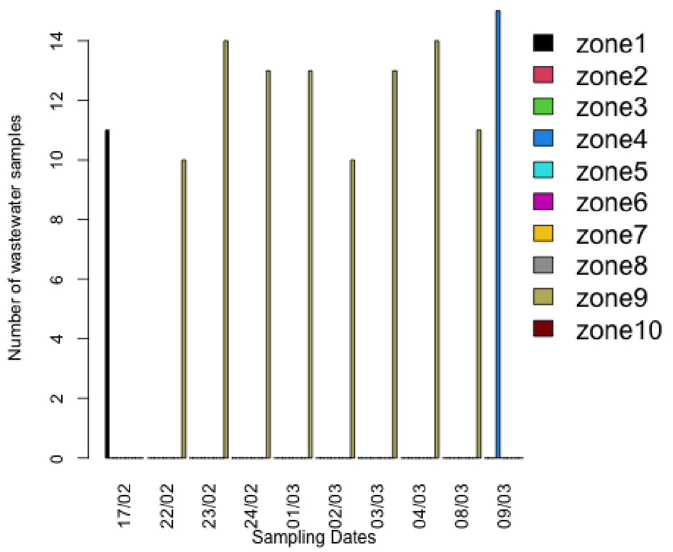

Figure S1C: Bar-plot of the number of wastewater samples collected within the 10 catchment zones at specific dates (10/03 — 20/04).

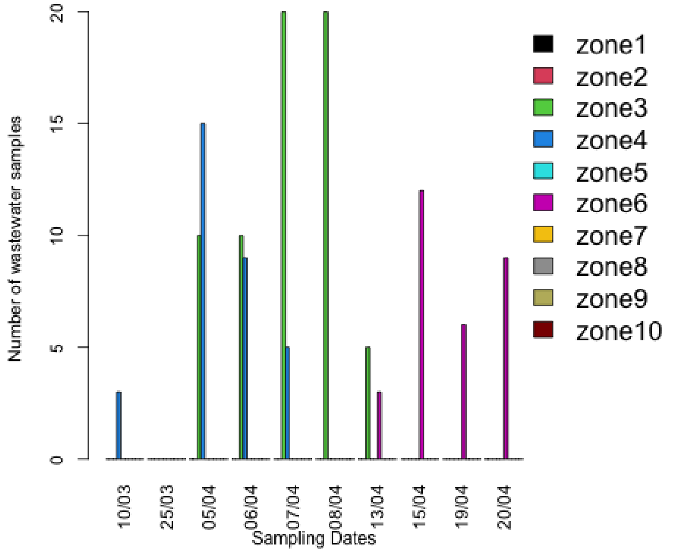

Figure S1D: Bar-plot of the number of wastewater samples collected within the 10 catchment zones at specific dates (21/04 — 06/05).

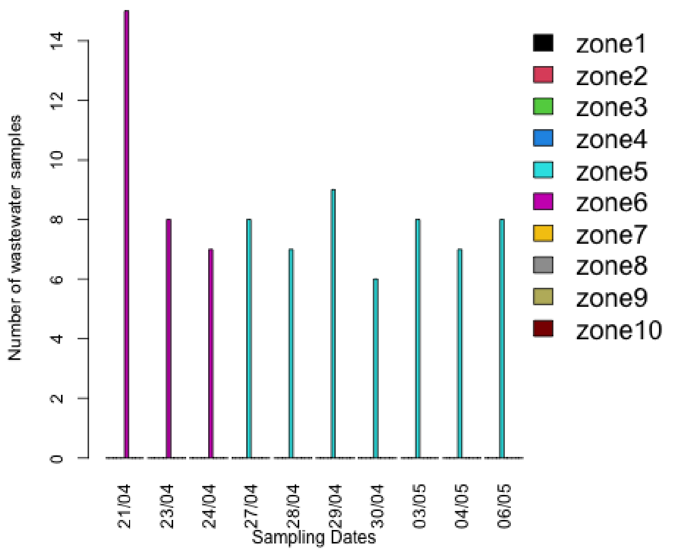

Figure S1E: Bar-plot of the number of wastewater samples collected within the 10 catchment zones at specific dates (07/05 — 02/07).

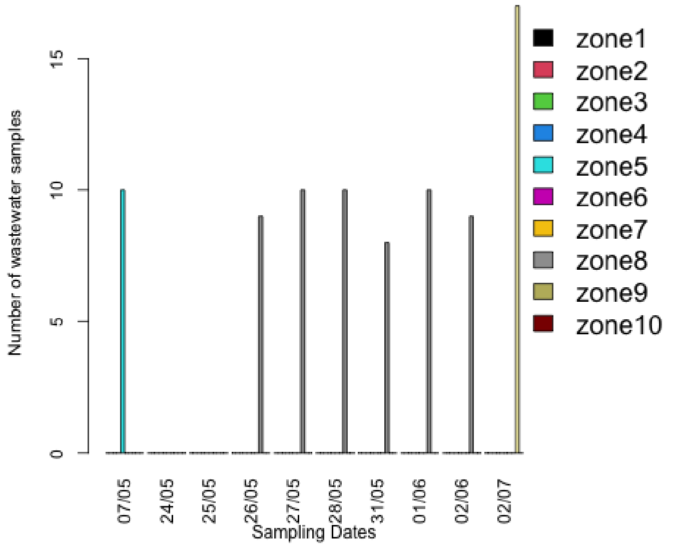

Figure S2: Histogram of the number of COVID-19 lab confirmed positives and mean SARS-CoV-2 log copy values of wastewater samples collected at specific dates.

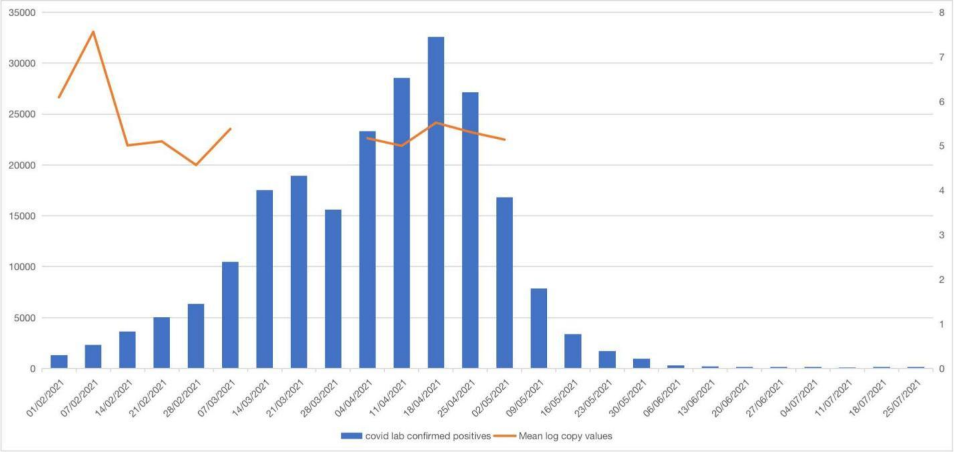

Figure S3A: Histogram and qq-plot of Temperature and Humidity embedded with p-value of Sharpiro-Wilk test of normality respectively for (a) urban and (b) rural.

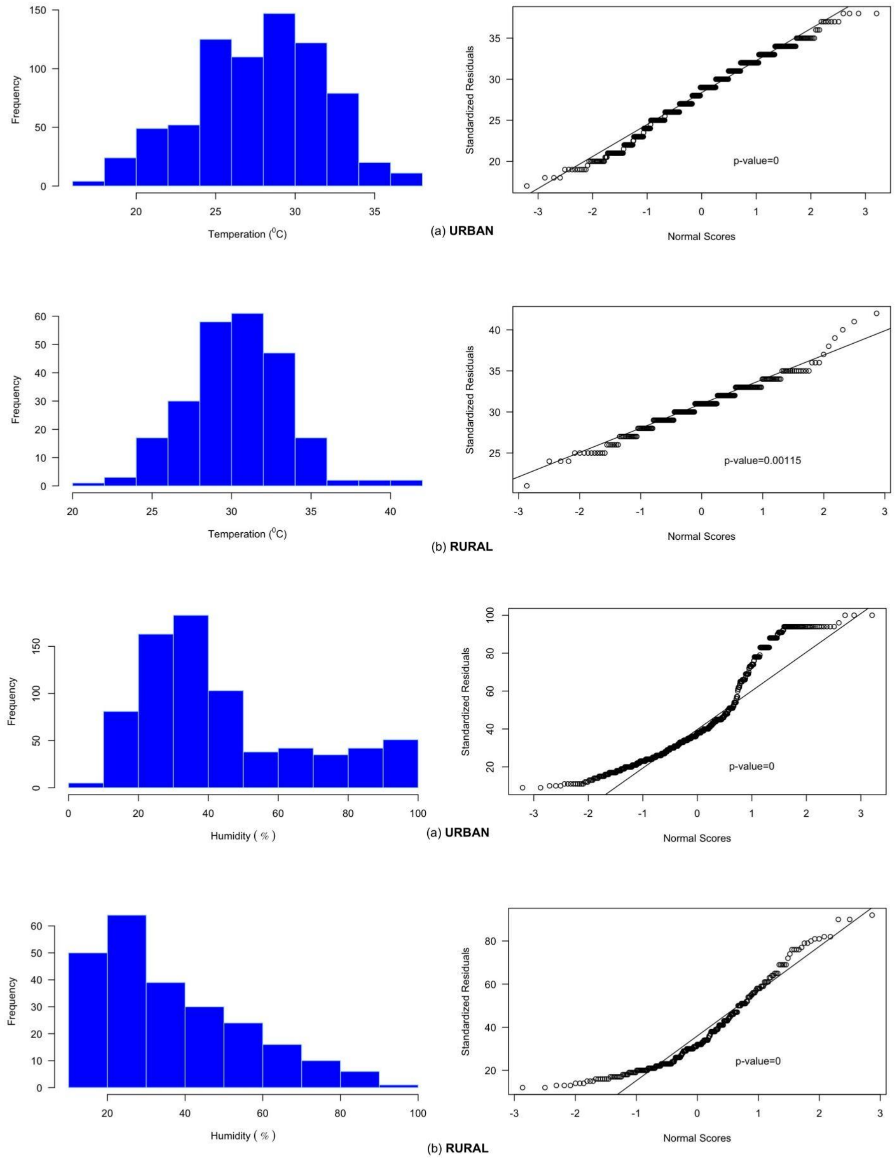

Figure S3B: Histogram and qq-plot of IC and E(ct) embedded with p− value of Sharpiro-Wilk test of normality respectively for (a) urban and (b) rural.

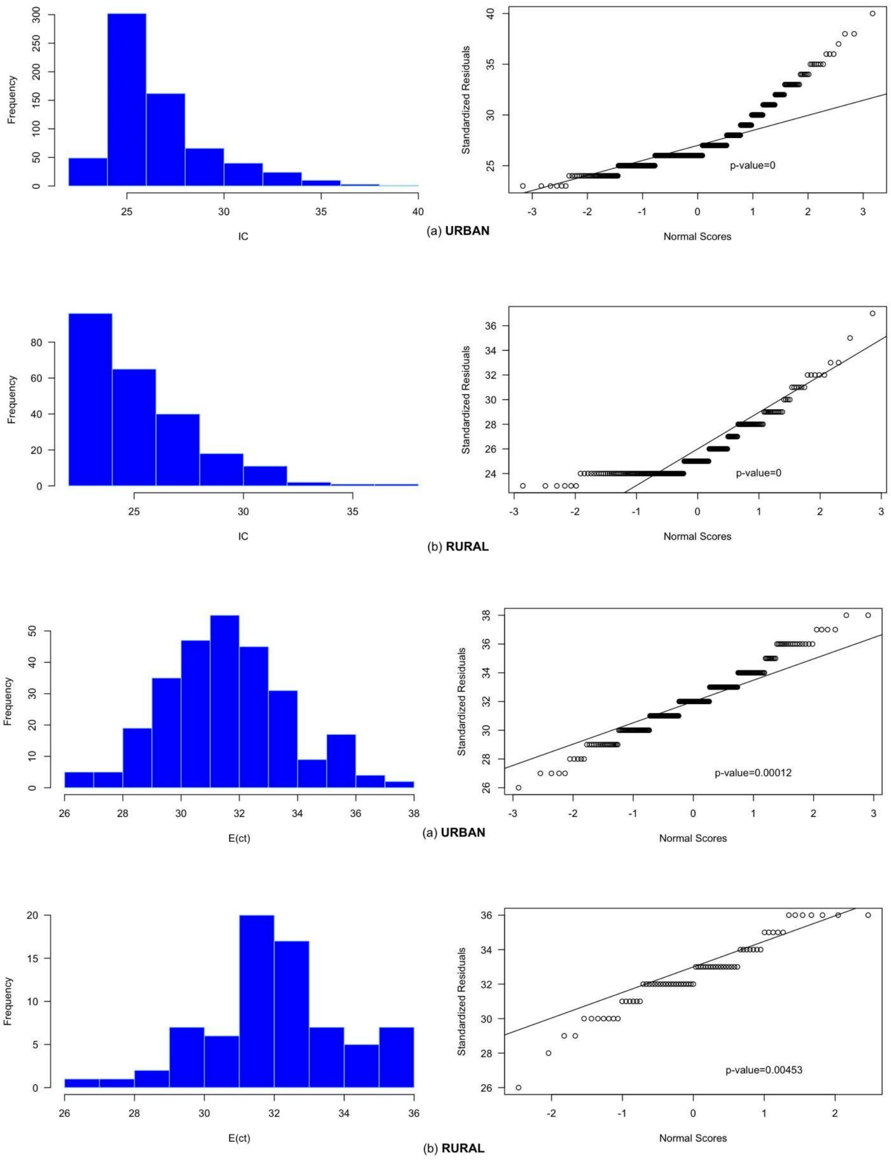

Figure S3C: Histogram and qq-plot of RdpdP (ct) and N (ct) embedded with p-value of Sharpiro-Wilk test of normality respectively for (a) urban and (b) rural.

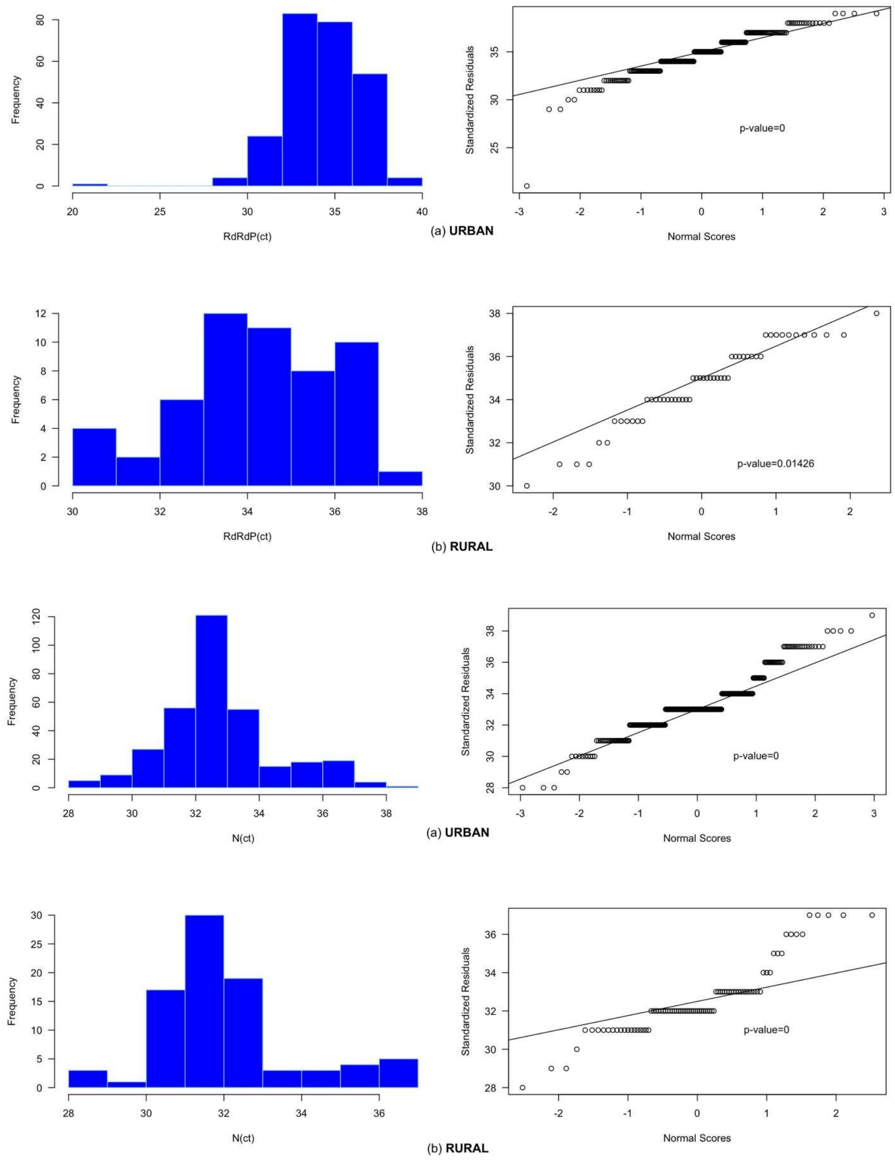

Figure S3D: Histogram and qq-plot of N gene (ct) and RNA (copies per L) embedded with p-value of Sharpiro-Wilk test of normality respectively for (a) urban and (b) rural.

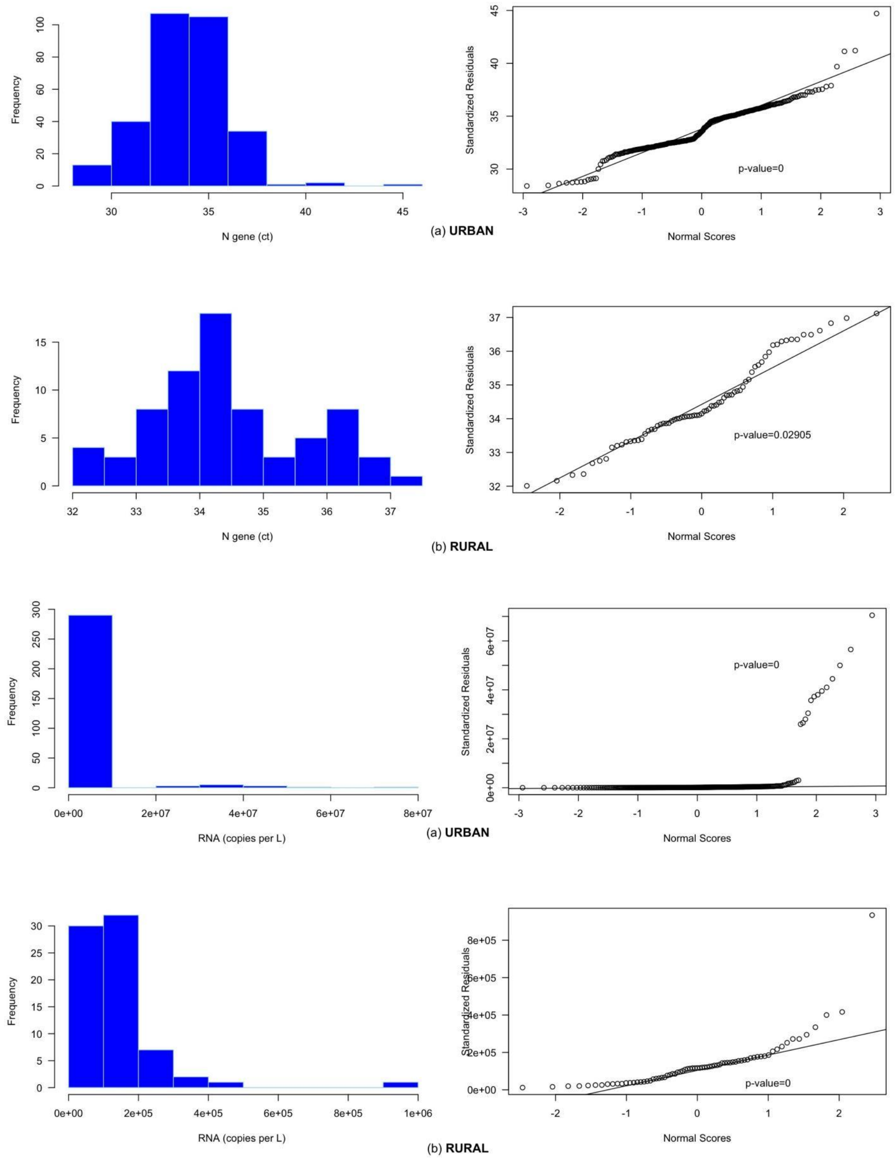

Figure S4A: Bar plots of RT-PCR results, Rainfall presence, Nearby Hospital, Sanitation, Population Density, Housing Condition, Drainage System and Livestock Domestication respectively for urban and rural.

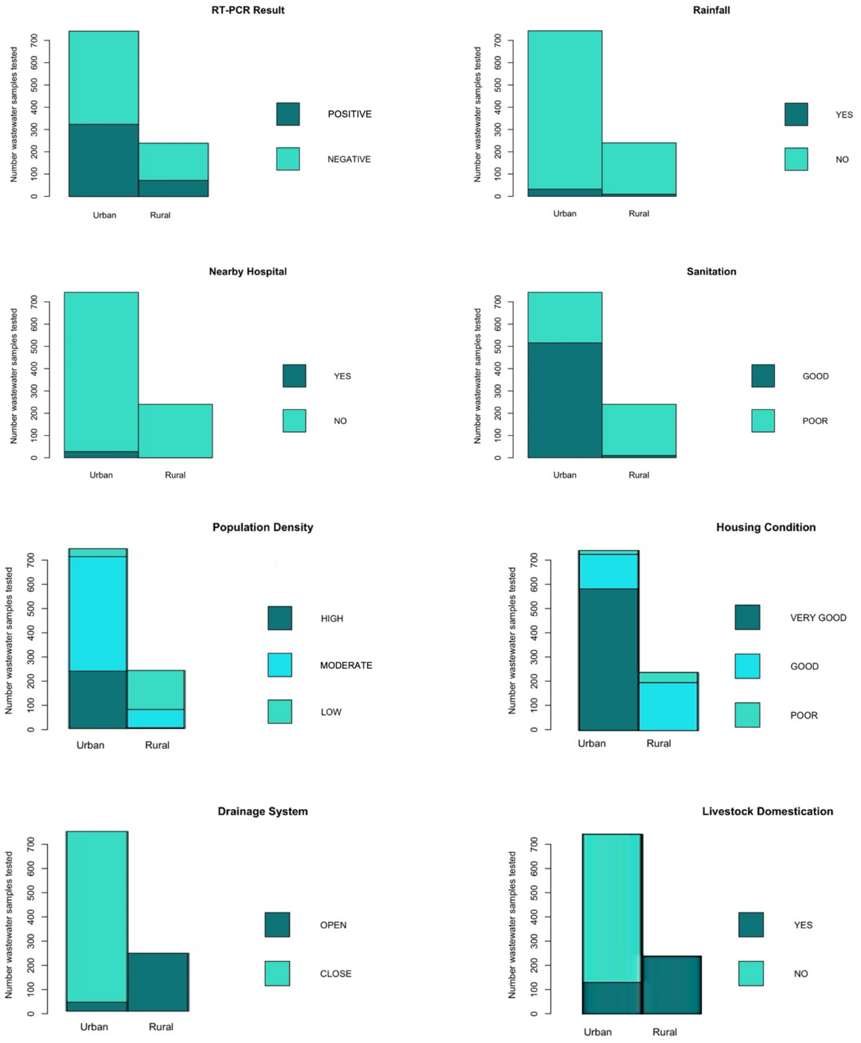

Figure S4B: Bar plots of Market Area, Industrial Area, Airport, Rail Station and Bus Station respectively for urban and rural.

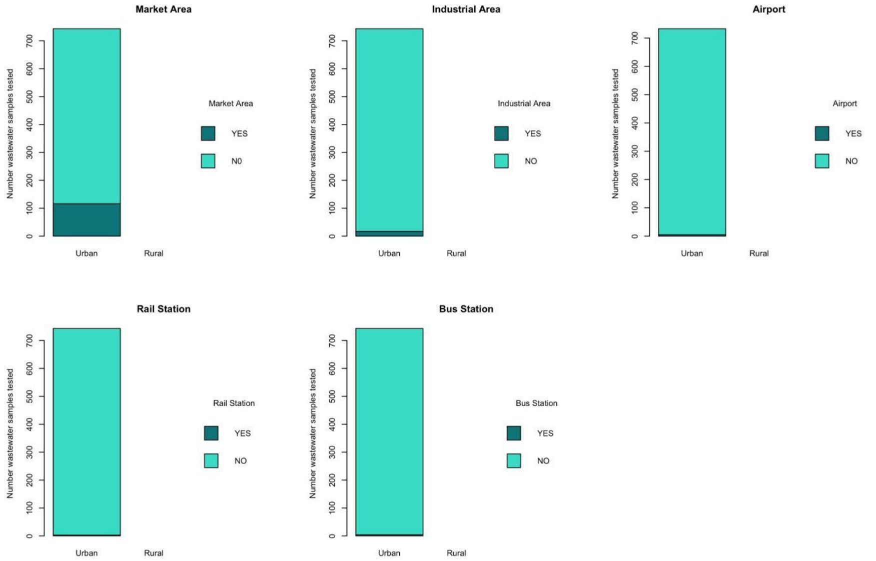

Figure S5: Data fitting to the proportions of the population that are: (left) confirmed positive of COVID-19 infection and (right) deaths via the SEIPR model (2.5), respectively for (a) Zone 1, (b) Zone 2, (c) Zone 7, and (d) Zone 9.

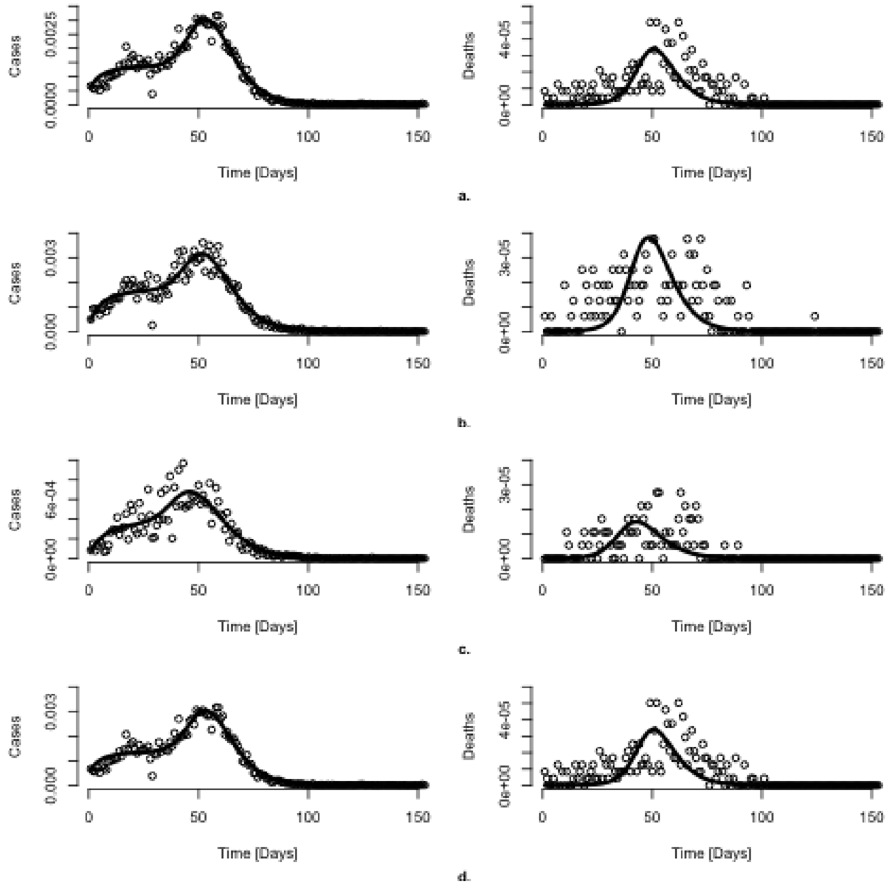

Figure S6A: Considering a viral half-life at an ambient temperature of the drainage of 30 h. Model fit to the proportion of the population that are (a) (left) confirmed positive COVID-19 infections and (b) (right) confirmed deaths from COVID-19 infections for zone 3. (c) SEIPR model (1) prediction for the mass rate of SARS-CoV-2 RNA in wastewater over time via Monte-Carlo simulation represented by black points. (d) Zoomed-in plot of the predicted number of active COVID-19 cases versus SARS-CoV-2 RNA mass rate with individual Monte-Carlo simulations represented by grey points; where 75% CI and 95% CI denoted by the green and red solid lines respectively. Coloured data points denote the measured RNA mass rates and estimated infectious individuals based on equation (8) as presented in Table 4 respectively for an assumed average per capita wastewater rates of 120 L per person per day (red solid points) and 135 L per person per day (blue solid points) for zone 3.

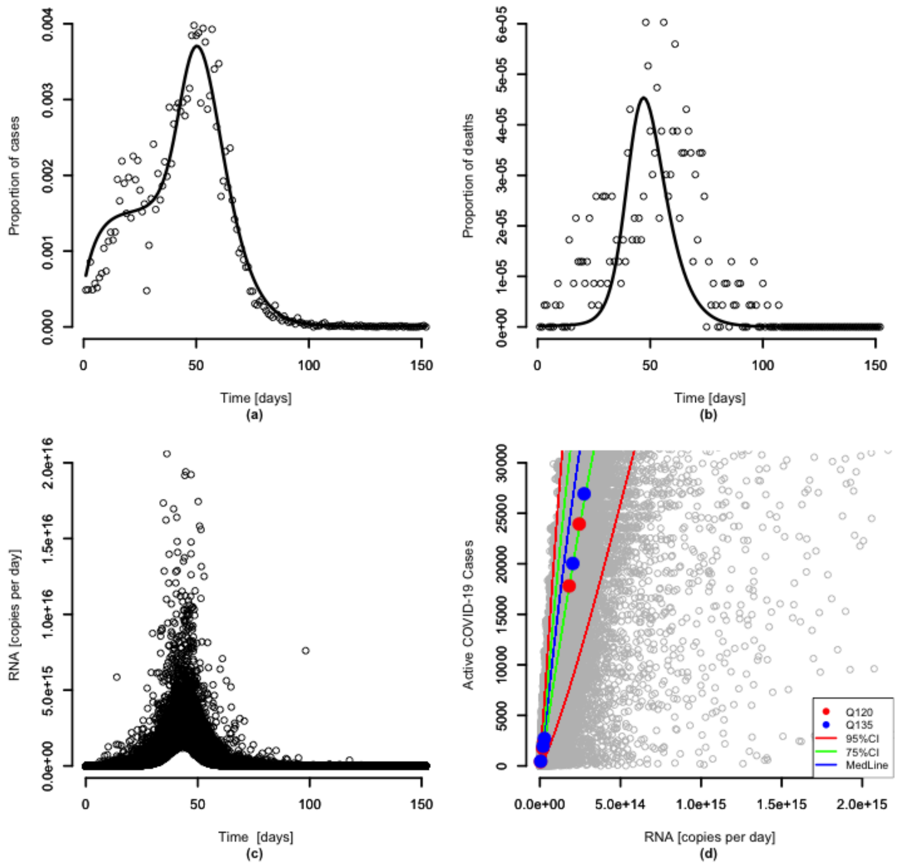

Figure S6B: Considering a viral half-life at an ambient temperature of the drainage of 30 h. Model fit to the proportion of the population that are (a) (left) confirmed positive COVID-19 infections and (b) (right) confirmed deaths from COVID-19 infections for zone 4. (c) SEIPR model (1) prediction for the mass rate of SARS-CoV-2 RNA in wastewater over time via Monte-Carlo simulation represented by black points. (d) Zoomed-in plot of the predicted number of active COVID-19 cases versus SARS-CoV-2 RNA mass rate with individual Monte-Carlo simulations represented by grey points; where 75% CI and 95% CI denoted by the green and red solid lines respectively. Coloured data points denote the measured RNA mass rates and estimated infectious individuals based on equation (8) as presented in Table 4 respectively for an assumed average per capita wastewater rates of 120 L per person per day (red solid points) and 135 L per person per day (blue solid points) for zone 4.

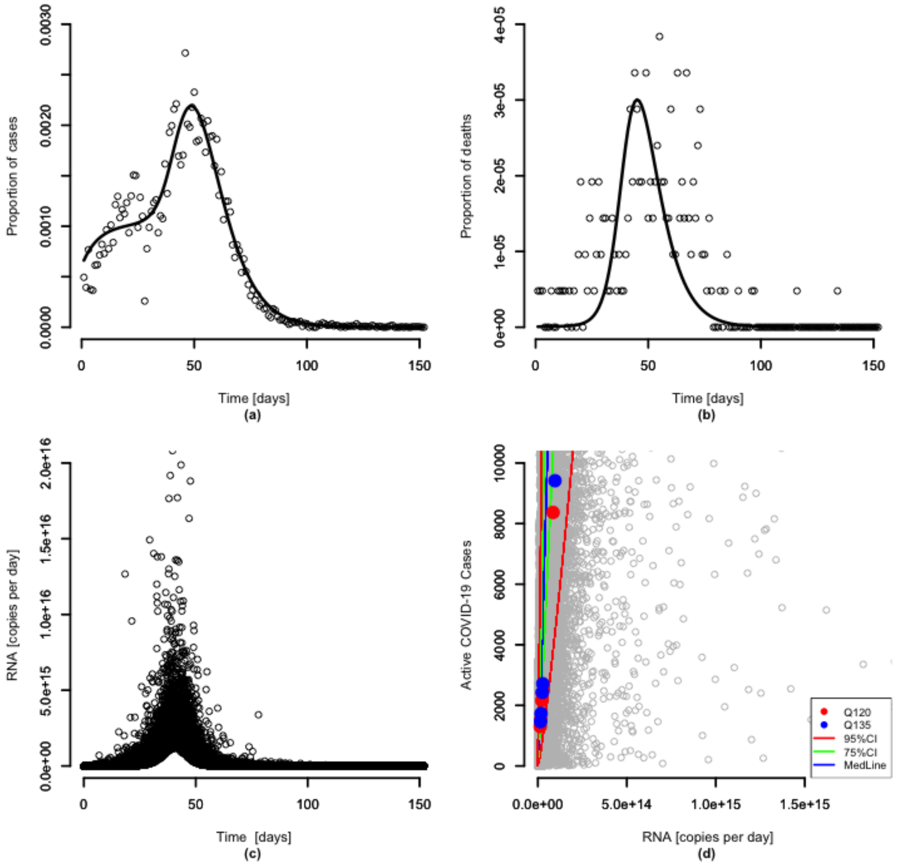

Figure S6C: Considering a viral half-life at an ambient temperature of the drainage of 30 h. Model fit to the proportion of the population that are (a) (left) confirmed positive COVID-19 infections and (b) (right) confirmed deaths from COVID-19 infections for zone 5. (c) SEIPR model (1) prediction for the mass rate of SARS-CoV-2 RNA in wastewater over time via Monte-Carlo simulation represented by black points. (d) Zoomed-in plot of the predicted number of active COVID-19 cases versus SARS-CoV-2 RNA mass rate with individual Monte-Carlo simulations represented by grey points; where 75% CI and 95% CI denoted by the green and red solid lines respectively. Coloured data points denote the measured RNA mass rates and estimated infectious individuals based on equation (8) as presented in Table 4 respectively for an assumed average per capita wastewater rates of 120 L per person per day (red solid points) and 135 L per person per day (blue solid points) for zone 5.

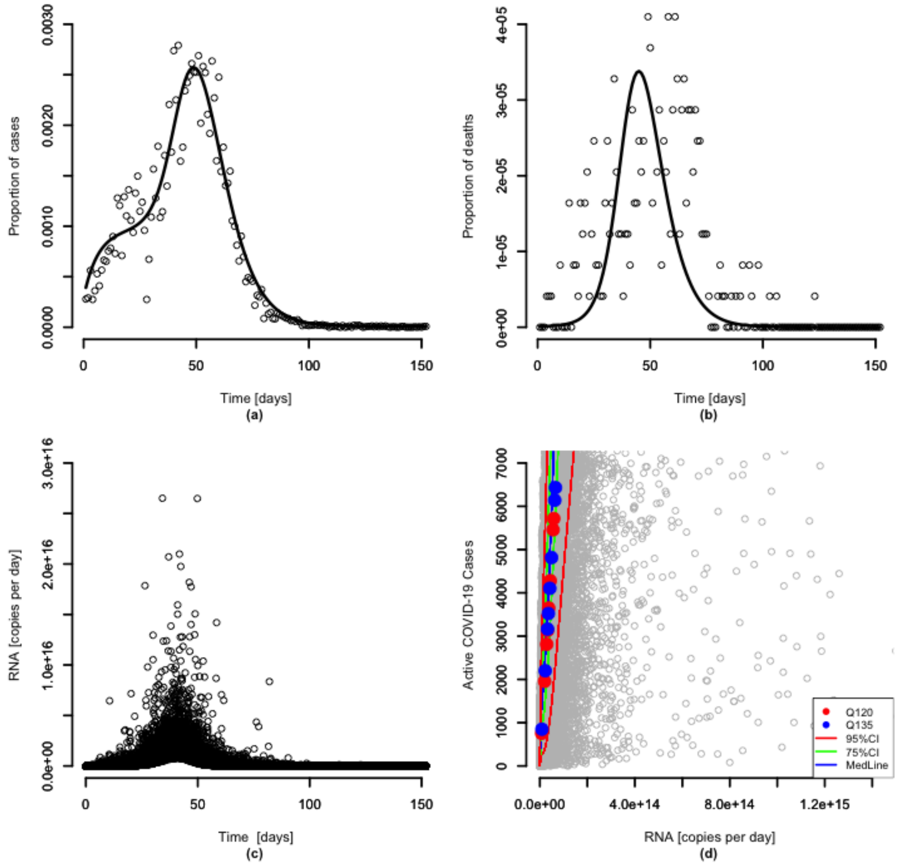

Figure S6D: Considering a viral half-life at an ambient temperature of the drainage of 30 h. Model fit to the proportion of the population that are (a) (left) confirmed positive COVID-19 infections and (b) (right) confirmed deaths from COVID-19 infections for zone 6. (c) SEIPR model (1) prediction for the mass rate of SARS-CoV-2 RNA in wastewater over time via Monte-Carlo simulation represented by black points. (d) Zoomed-in plot of the predicted number of active COVID-19 cases versus SARS-CoV-2 RNA mass rate with individual Monte-Carlo simulations represented by grey points; where 75% CI and 95% CI denoted by the green and red solid lines respectively. Coloured data points denote the measured RNA mass rates and estimated infectious individuals based on equation (8) as presented in Table 4 respectively for an assumed average per capita wastewater rates of 120 L per person per day (red solid points) and 135 L per person per day (blue solid points) for zone 6.

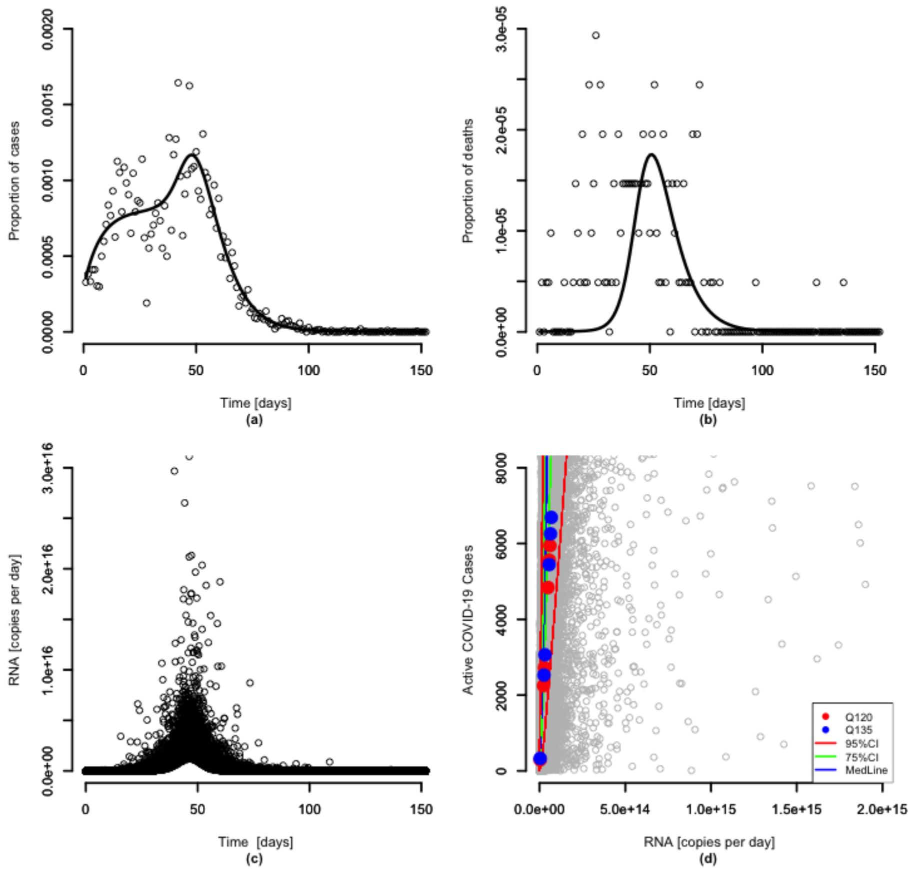

Figure S6E: Considering a viral half-life at an ambient temperature of the drainage of 30 h. Model fit to the proportion of the population that are (a) (left) confirmed positive COVID-19 infections and (b) (right) confirmed deaths from COVID-19 infections for zone 8. (c) SEIPR model (1) prediction for the mass rate of SARS-CoV-2 RNA in wastewater over time via Monte-Carlo simulation represented by black points. (d) Zoomed-in plot of the predicted number of active COVID-19 cases versus SARS-CoV-2 RNA mass rate with individual Monte-Carlo simulations represented by grey points; where 75% CI and 95% CI denoted by the green and red solid lines respectively. Coloured data points denote the measured RNA mass rates and estimated infectious individuals based on equation (8) as presented in Table 4 respectively for an assumed average per capita wastewater rates of 120 L per person per day (red solid points) and 135 L per person per day (blue solid points) for zone 8.

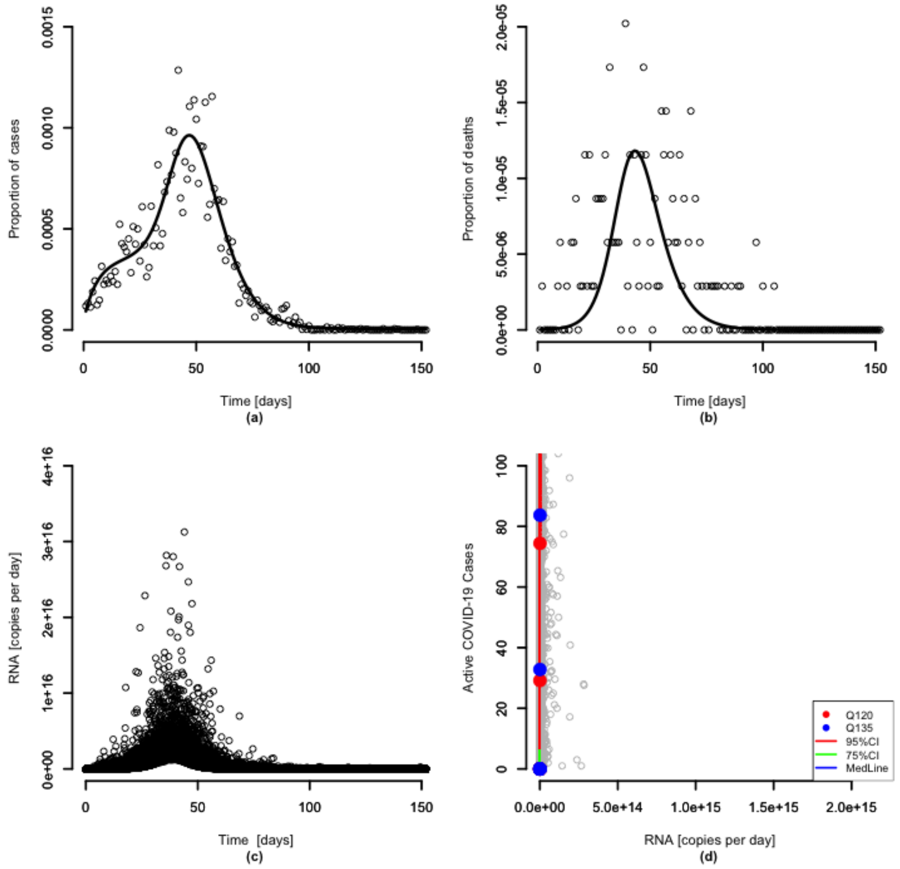

Figure S6F: Considering a viral half-life at an ambient temperature of the drainage of 30 h. Model fit to the proportion of the population that are (a) (left) confirmed positive COVID-19 infections and (b) (right) confirmed deaths from COVID-19 infections for zone 10. (c) SEIPR model (1) prediction for the mass rate of SARS-CoV-2 RNA in wastewater over time via Monte-Carlo simulation represented by black points. (d) Zoomed-in plot of the predicted number of active COVID-19 cases versus SARS-CoV-2 RNA mass rate with individual Monte-Carlo simulations represented by grey points; where 75% CI and 95% CI denoted by the green and red solid lines respectively. Coloured data points denote the measured RNA mass rates and estimated infectious individuals based on equation (8) as presented in Table 4 respectively for an assumed average per capita wastewater rates of 120 L per person per day (red solid points) and 135 L per person per day (blue solid points) for zone 10.

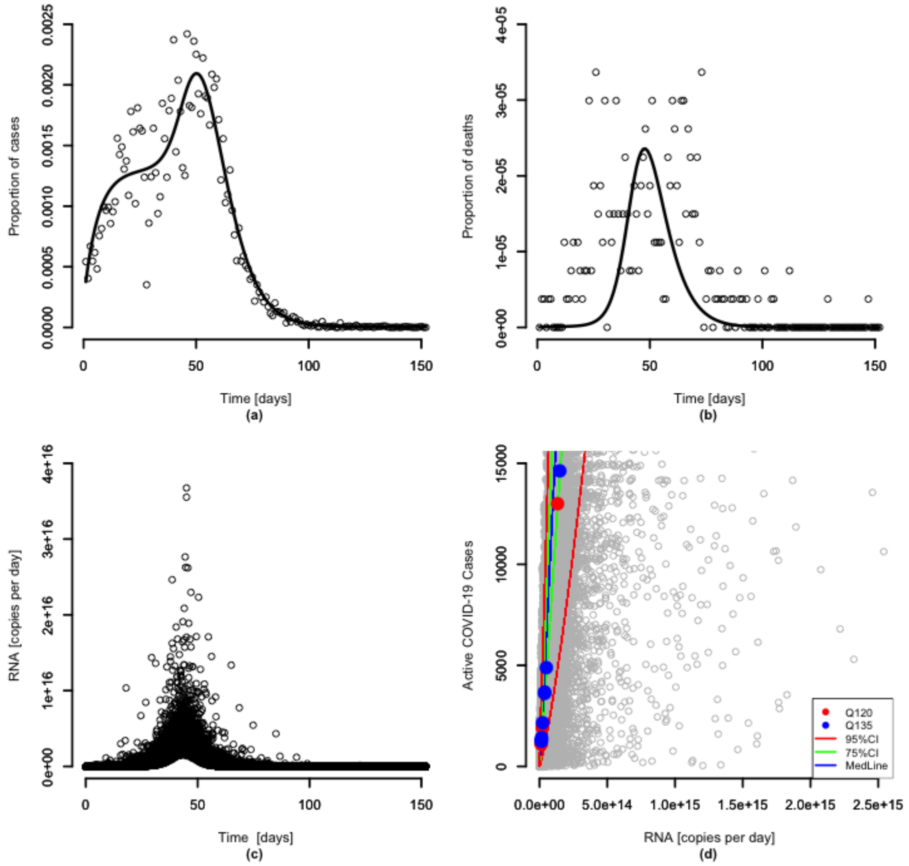

Figure S7A: Considering a viral half-life at an ambient temperature of the drainage of 3 h. Model fit of the proportion of the population that are (a) (left) confirmed positive COVID-19 infections and (b) (right) confirmed deaths from COVID-19 infections for all zones as a single unit. (c) SEIPR model (1) prediction for the mass rate of SARS-CoV-2 RNA in wastewater over time via Monte-Carlo simulation represented by black points. (d) Zoomed-in plot of the predicted number of active COVID-19 cases versus SARS-CoV-2 RNA mass rate with individual Monte-Carlo simulations represented by grey points; where 75% CI and 95% CI denoted by the green and red solid lines respectively. Coloured data points denote the measured RNA mass rates and estimated infectious individuals based on equation (8) as presented in Table 4 respectively for an assumed average per capita wastewater rates of 120 L per person per day (red solid points) and 135 L per person per day (blue solid points) for all zones as a single unit.

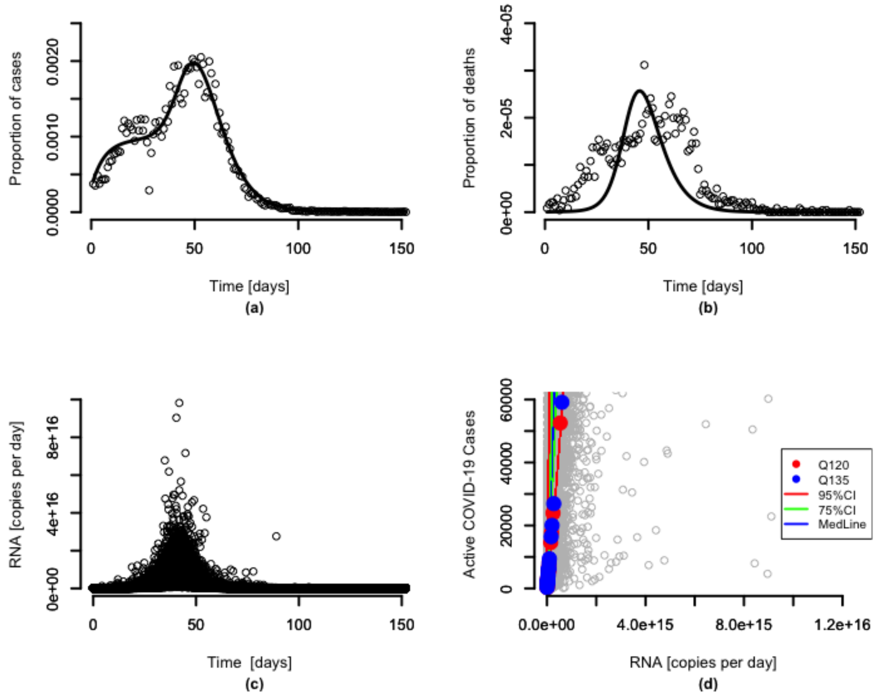

Figure S7B: Considering a viral half-life at an ambient temperature of the drainage of 3 h. Model fit to the proportion of the population that are (a) (left) confirmed positive COVID-19 infections and (b) (right) confirmed deaths from COVID-19 infections for zone 3. (c) SEIPR model (1) prediction for the mass rate of SARS-CoV-2 RNA in wastewater over time via Monte-Carlo simulation represented by black points. (d) Zoomed-in plot of the predicted number of active COVID-19 cases versus SARS-CoV-2 RNA mass rate with individual Monte-Carlo simulations represented by grey points; where 75% CI and 95% CI denoted by the green and red solid lines respectively. Coloured data points denote the measured RNA mass rates and estimated infectious individuals based on equation (8) as presented in Table 4 respectively for an assumed average per capita wastewater rates of 120 L per person per day (red solid points) and 135 L per person per day (blue solid points) for zone 3.

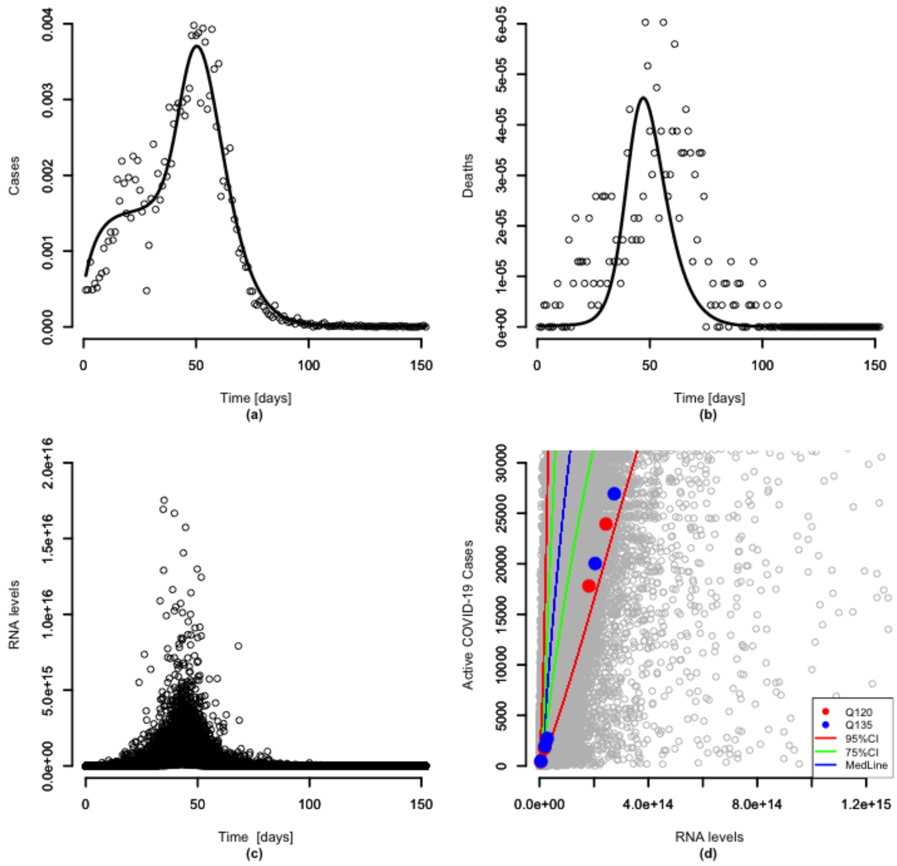

Figure S7C: Considering a viral half-life at an ambient temperature of the drainage of 3 h. Model fit to the proportion of the population that are (a) (left) confirmed positive COVID-19 infections and (b) (right) confirmed deaths from COVID-19 infections for zone 4. (c) SEIPR model (1) prediction for the mass rate of SARS-CoV-2 RNA in wastewater over time via Monte-Carlo simulation represented by black points. (d) Zoomed-in plot of the predicted number of active COVID-19 cases versus SARS-CoV-2 RNA mass rate with individual Monte-Carlo simulations represented by grey points; where 75% CI and 95% CI denoted by the green and red solid lines respectively. Coloured data points denote the measured RNA mass rates and estimated infectious individuals based on equation (8) as presented in Table 4 respectively for an assumed average per capita wastewater rates of 120 L per person per day (red solid points) and 135 L per person per day (blue solid points) for zone 4.

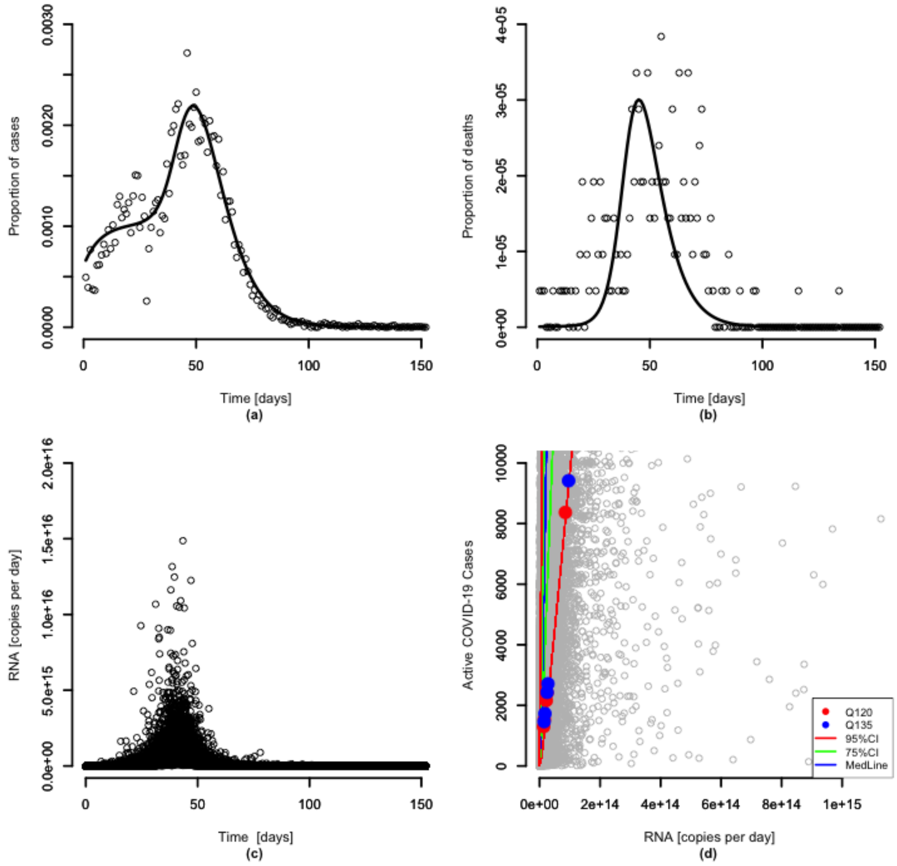

Figure S7D: Considering a viral half-life at an ambient temperature of the drainage of 3 h. Model fit to the proportion of the population that are (a) (left) confirmed positive COVID-19 infections and (b) (right) confirmed deaths from COVID-19 infections for zone 5. (c) SEIPR model (1) prediction for the mass rate of SARS-CoV-2 RNA in wastewater over time via Monte-Carlo simulation represented by black points. (d) Zoomed-in plot of the predicted number of active COVID-19 cases versus SARS-CoV-2 RNA mass rate with individual Monte-Carlo simulations represented by grey points; where 75% CI and 95% CI denoted by the green and red solid lines respectively. Coloured data points denote the measured RNA mass rates and estimated infectious individuals based on equation (8) as presented in Table 4 respectively for an assumed average per capita wastewater rates of 120 L per person per day (red solid points) and 135 L per person per day (blue solid points) for zone 5.

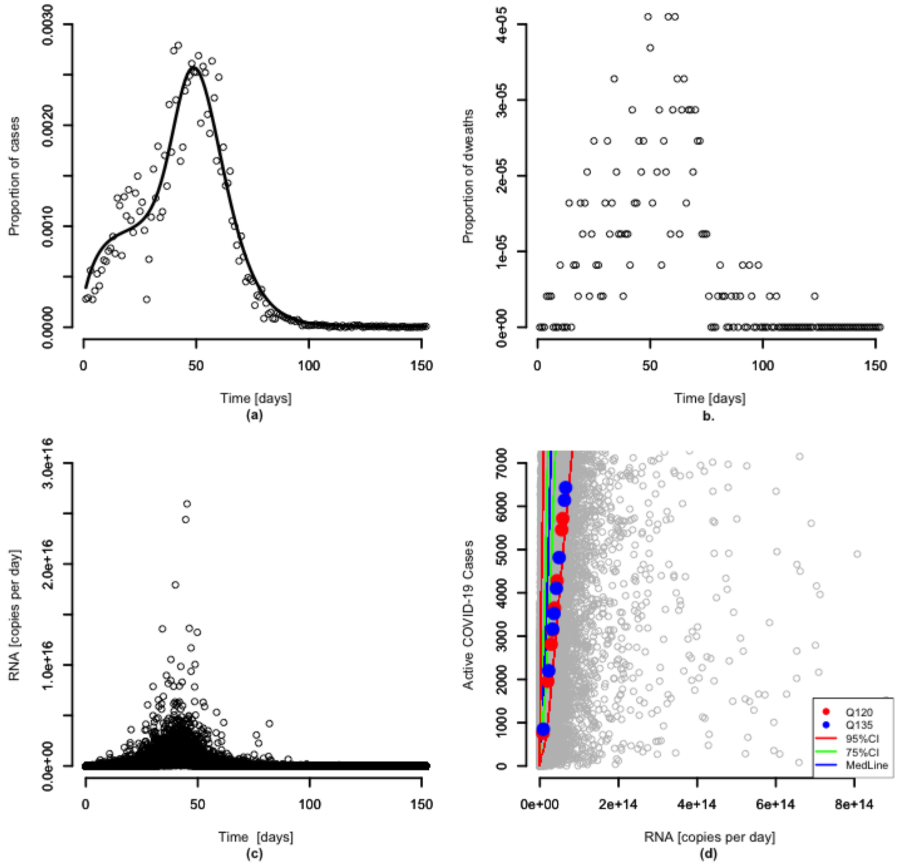

Figure S7E: Considering a viral half-life at an ambient temperature of the drainage of 3 h. Model fit to the proportion of the population that are (a) (left) confirmed positive COVID-19 infections and (b) (right) confirmed deaths from COVID-19 infections for zone 6. (c) SEIPR model (1) prediction for the mass rate of SARS-CoV-2 RNA in wastewater over time via Monte-Carlo simulation represented by black points. (d) Zoomed-in plot of the predicted number of active COVID-19 cases versus SARS-CoV-2 RNA mass rate with individual Monte-Carlo simulations represented by grey points; where 75% CI and 95% CI denoted by the green and red solid lines respectively. Coloured data points denote the measured RNA mass rates and estimated infectious individuals based on equation (8) as presented in Table 4 respectively for an assumed average per capita wastewater rates of 120 L per person per day (red solid points) and 135 L per person per day (blue solid points) for zone 6.

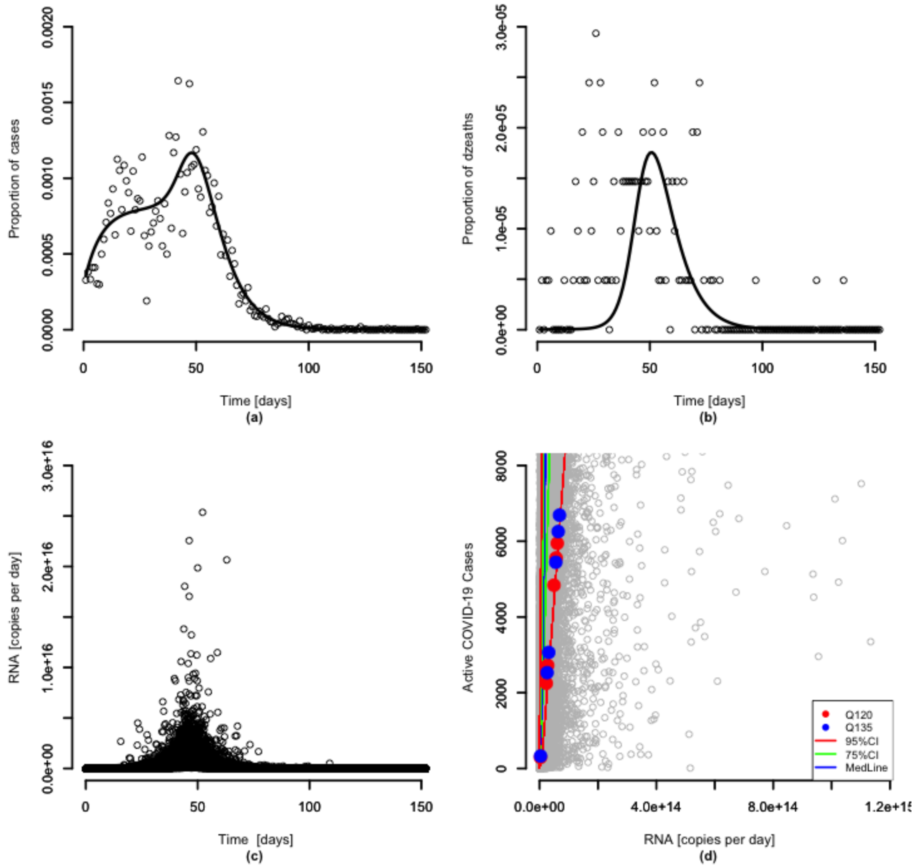

Figure S7F: Considering a viral half-life at an ambient temperature of the drainage of 3 h. Model fit to the proportion of the population that are (a) (left) confirmed positive COVID-19 infections and (b) (right) confirmed deaths from COVID-19 infections for zone 8. (c) SEIPR model (1) prediction for the mass rate of SARS-CoV-2 RNA in wastewater over time via Monte-Carlo simulation represented by black points. (d) Zoomed-in plot of the predicted number of active COVID-19 cases versus SARS-CoV-2 RNA mass rate with individual Monte-Carlo simulations represented by grey points; where 75% CI and 95% CI denoted by the green and red solid lines respectively. Coloured data points denote the measured RNA mass rates and estimated infectious individuals based on equation (8) as presented in Table 4 respectively for an assumed average per capita wastewater rates of 120 L per person per day (red solid points) and 135 L per person per day (blue solid points) for zone 8.

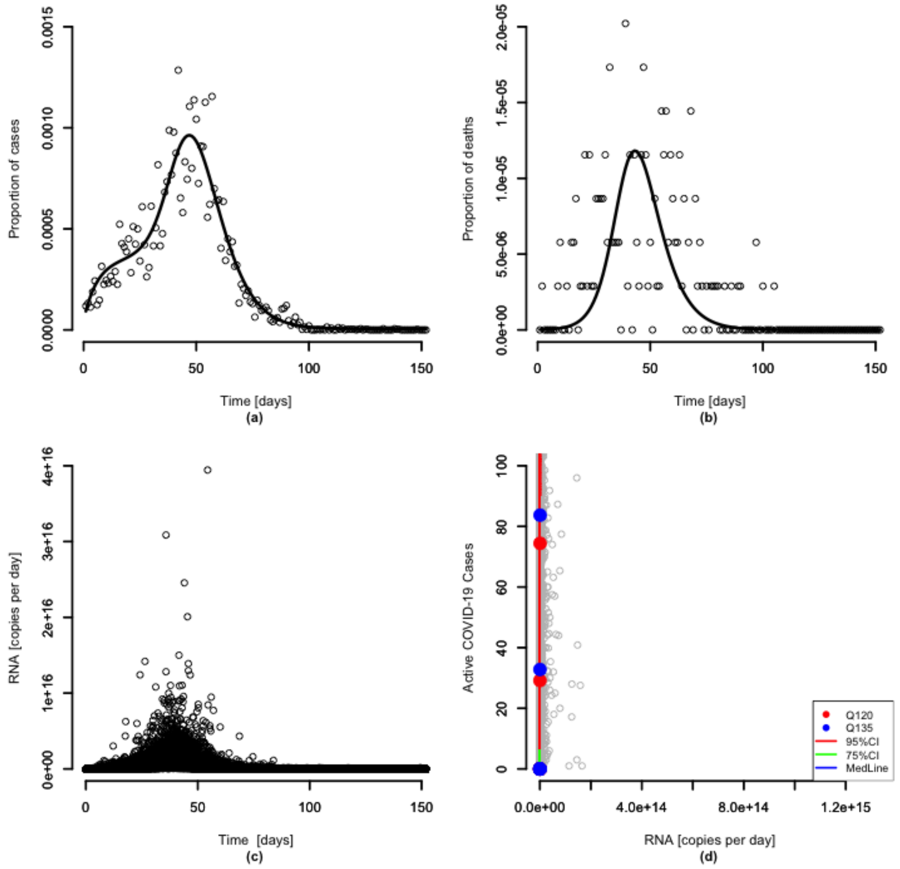

Figure S7G: Considering a viral half-life at an ambient temperature of the drainage of 3 h. Model fit to the proportion of the population that are (a) (left) confirmed positive COVID-19 infections and (b) (right) confirmed deaths from COVID-19 infections for zone 10. (c) SEIPR model (1) prediction for the mass rate of SARS-CoV-2 RNA in wastewater over time via Monte-Carlo simulation represented by black points. (d) Zoomed-in plot of the predicted number of active COVID-19 cases versus SARS-CoV-2 RNA mass rate with individual Monte-Carlo simulations represented by grey points; where 75% CI and 95% CI denoted by the green and red solid lines respectively. Coloured data points denote the measured RNA mass rates and estimated infectious individuals based on equation (8) as presented in Table 4 respectively for an assumed average per capita wastewater rates of 120 L per person per day (red solid points) and 135 L per person per day (blue solid points) for zone 10.

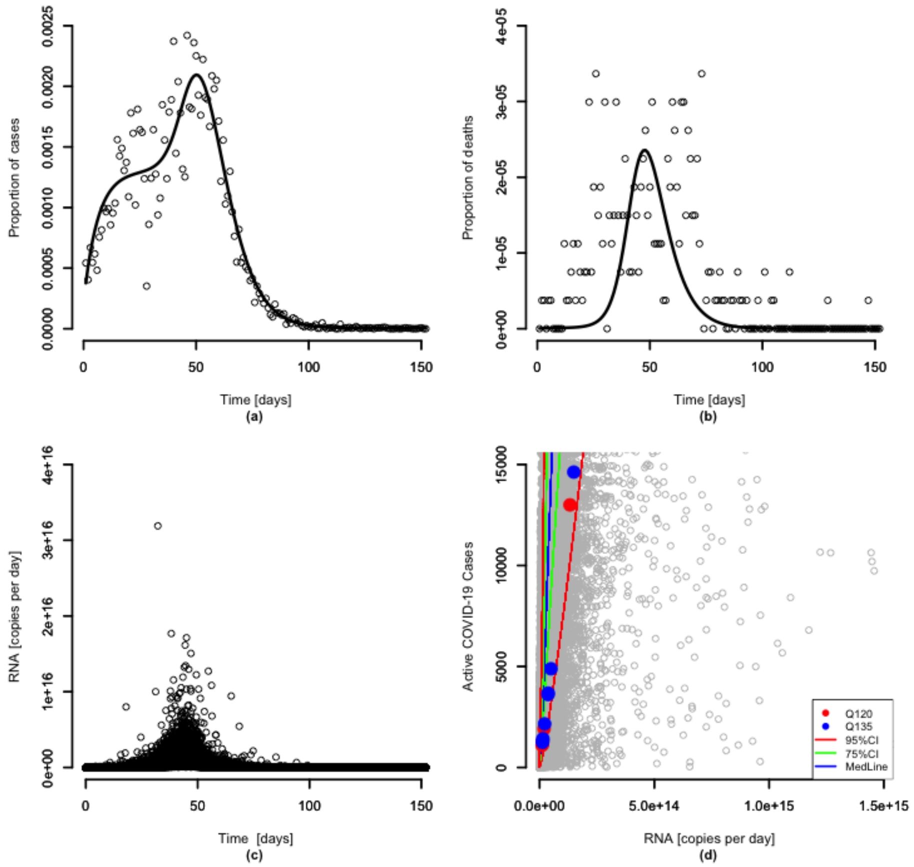

Figure S8: : Linear relationship between (a) R_0 and temperature, (b) R_0 and humidity (c) R_0 and population density, (d) β_1 and temperature, (e) β_1 and population density.

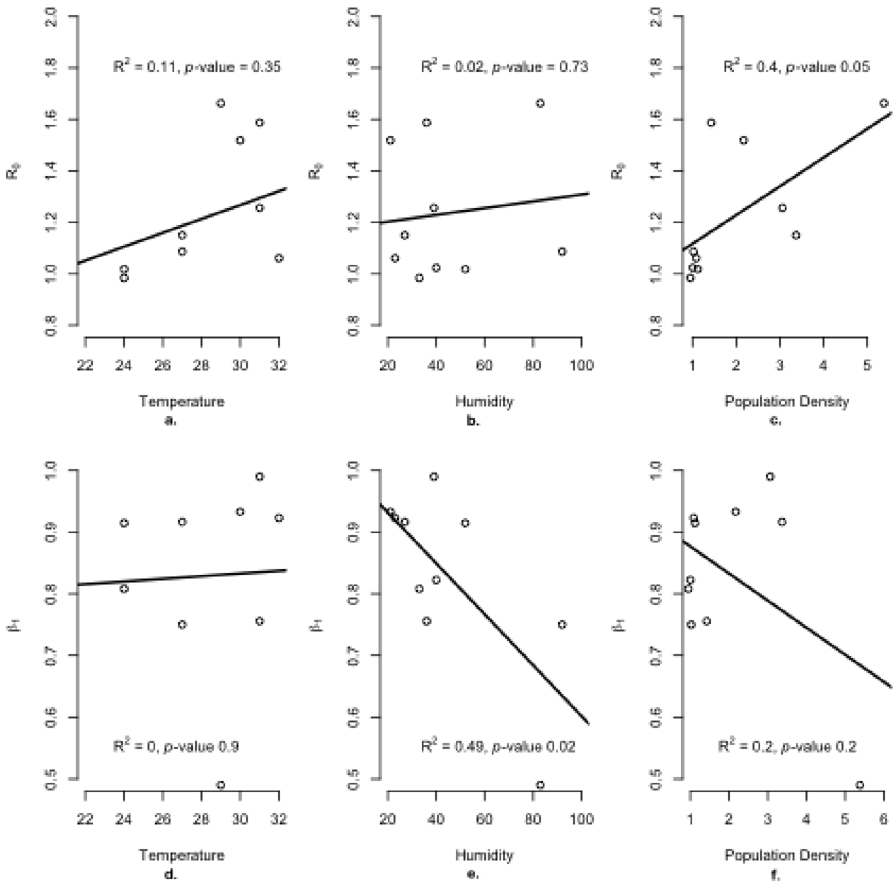

Figure S9A: Considering a viral half-life at an ambient temperature of the drainage of 30 h. Model fit to the proportion of the population that are (a) (left) confirmed positive COVID-19 infections and (b) (right) confirmed deaths from COVID-19 infections for all zones as a single unit. (c) SEIPR model (1) prediction for the mass rate of SARS-CoV-2 RNA in wastewater over time via Monte-Carlo simulation represented by black points. (d) Plot of the predicted number of active COVID-19 cases versus SARS-CoV-2 RNA mass rate with individual Monte-Carlo simulations represented by grey points; where 75% CI and 95% CI are denoted by the green and red solid lines respectively. Coloured data points denote the measured RNA mass rates and estimated infectious individuals based on equation (8) as presented in Table 4 respectively for an assumed average per capita wastewater rates of 120 L per person per day (red solid points) and 135 L per person per day (blue solid points) for all zones as a single unit.

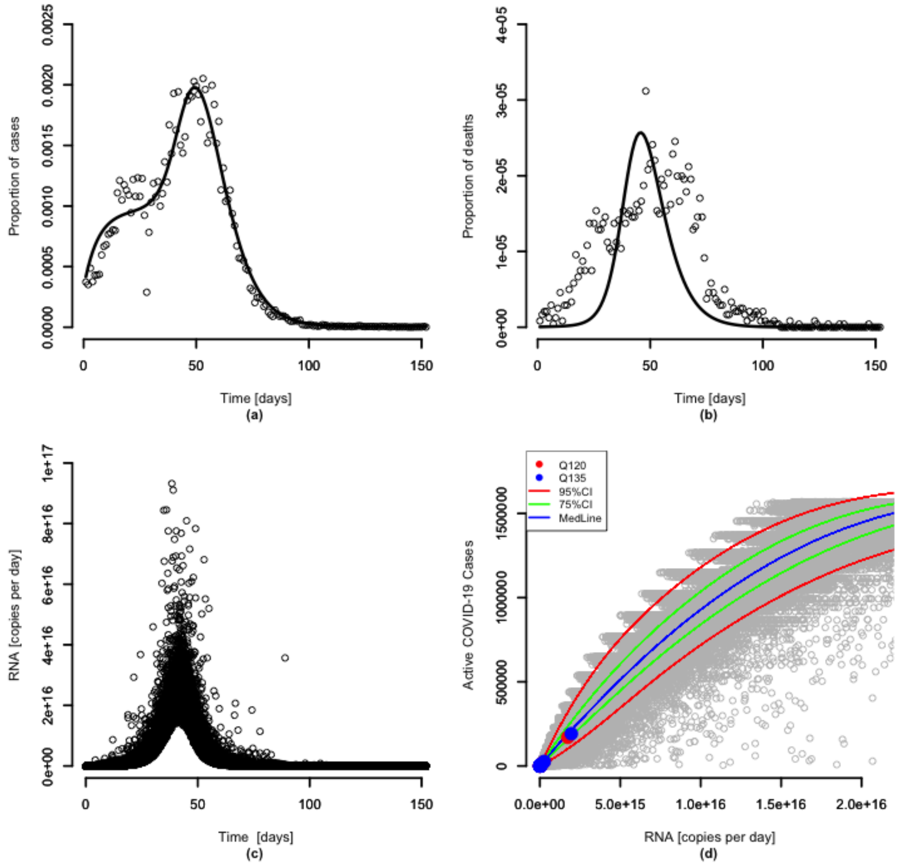

Figure S9B: Considering a viral half-life at an ambient temperature of the drainage of 30 h. Model fit to the proportion of the population that are (a) (left) confirmed positive COVID-19 infections and (b) (right) confirmed deaths from COVID-19 infections for zone 3. (c) SEIPR model (1) prediction for the mass rate of SARS-CoV-2 RNA in wastewater over time via Monte-Carlo simulation represented by black points. (d) Plot of the predicted number of active COVID-19 cases versus SARS-CoV-2 RNA mass rate with individual Monte-Carlo simulations represented by grey points; where 75% CI and 95% CI are denoted by the green and red solid lines respectively. Coloured data points denote the measured RNA mass rates and estimated infectious individuals based on equation (8) as presented in Table 4 respectively for an assumed average per capita wastewater rates of 120 L per person per day (red solid points) and 135 L per person per day (blue solid points) for zone 3.

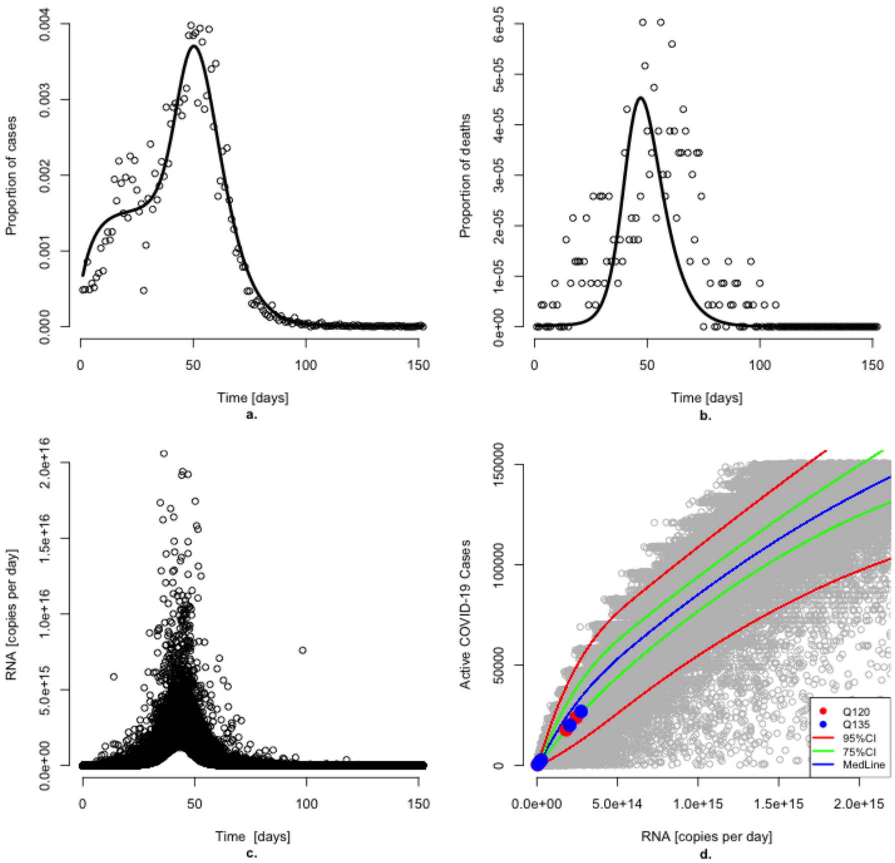

Figure S9C: Considering a viral half-life at an ambient temperature of the drainage of 30 h. Model fit to the proportion of the population that are (a) (left) confirmed positive COVID-19 infections and (b) (right) confirmed deaths from COVID-19 infections for zone 4. (c) SEIPR model (1) prediction for the mass rate of SARS-CoV-2 RNA in wastewater over time via Monte-Carlo simulation represented by black points. (d) Plot of the predicted number of active COVID-19 cases versus SARS-CoV-2 RNA mass rate with individual Monte-Carlo simulations represented by grey points; where 75% CI and 95% CI are denoted by the green and red solid lines respectively. Coloured data points denote the measured RNA mass rates and estimated infectious individuals based on equation (8) as presented in Table 4 respectively for an assumed average per capita wastewater rates of 120 L per person per day (red solid points) and 135 L per person per day (blue solid points) for zone 4.

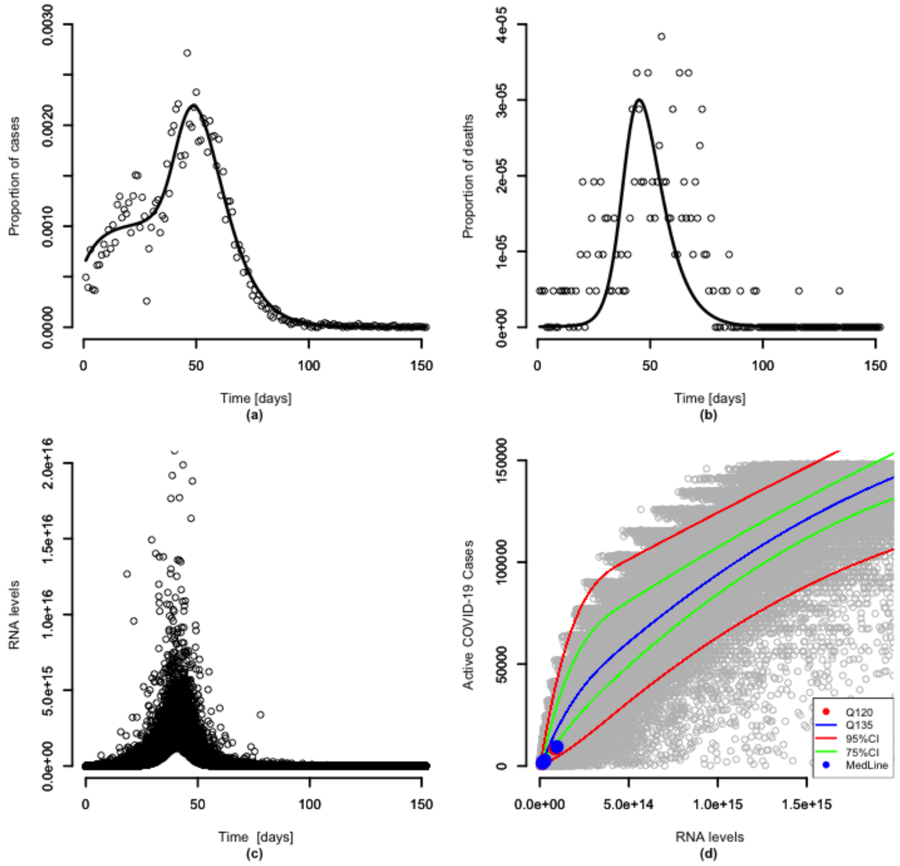

Figure S9D: Considering a viral half-life at an ambient temperature of the drainage of 30 h. Model fit to the proportion of the population that are (a) (left) confirmed positive COVID-19 infections and (b) (right) confirmed deaths from COVID-19 infections for zone 5. (c) SEIPR model (1) prediction for the mass rate of SARS-CoV-2 RNA in wastewater over time via Monte-Carlo simulation represented by black points. (d) Plot of the predicted number of active COVID-19 cases versus SARS-CoV-2 RNA mass rate with individual Monte-Carlo simulations represented by grey points; where 75% CI and 95% CI are denoted by the green and red solid lines respectively. Coloured data points denote the measured RNA mass rates and estimated infectious individuals based on equation (8) as presented in Table 4 respectively for an assumed average per capita wastewater rates of 120 L per person per day (red solid points) and 135 L per person per day (blue solid points) for zone 5.

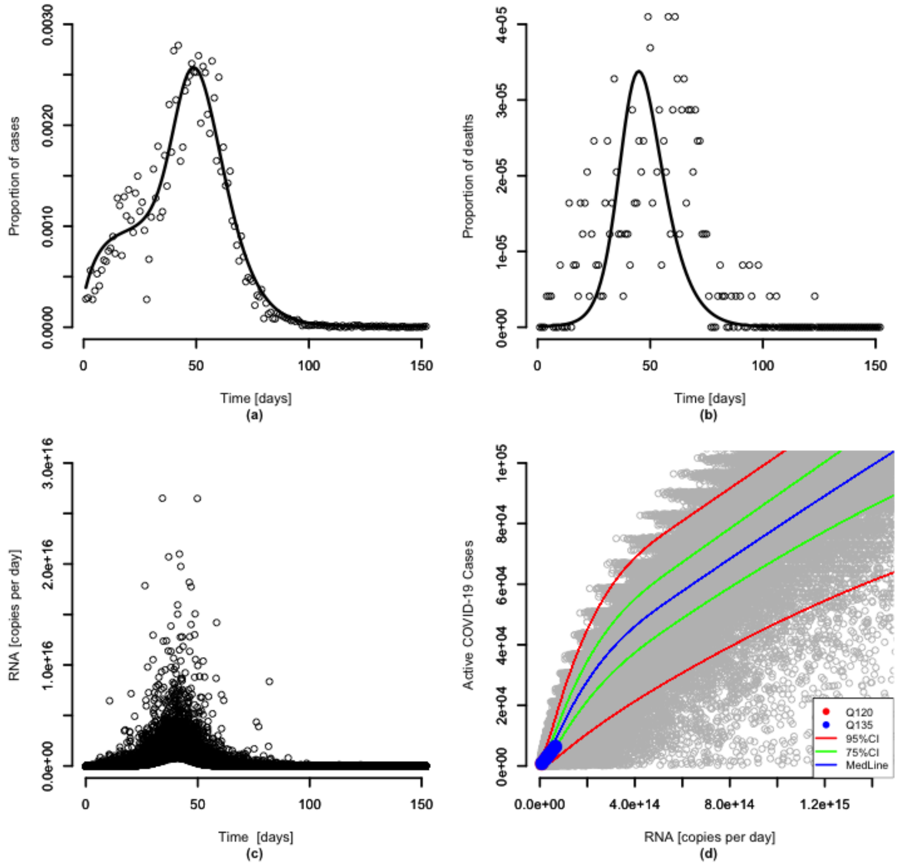

Figure S9E: Considering a viral half-life at an ambient temperature of the drainage of 30 h. Model fit to the proportion of the population that are (a) (left) confirmed positive COVID-19 infections and (b) (right) confirmed deaths from COVID-19 infections for zone 6. (c) SEIPR model (1) prediction for the mass rate of SARS-CoV-2 RNA in wastewater over time via Monte-Carlo simulation represented by black points. (d) Plot of the predicted number of active COVID-19 cases versus SARS-CoV-2 RNA mass rate with individual Monte-Carlo simulations represented by grey points; where 75% CI and 95% CI are denoted by the green and red solid lines respectively. Coloured data points denote the measured RNA mass rates and estimated infectious individuals based on equation (8) as presented in Table 4 respectively for an assumed average per capita wastewater rates of 120 L per person per day (red solid points) and 135 L per person per day (blue solid points) for zone 6.

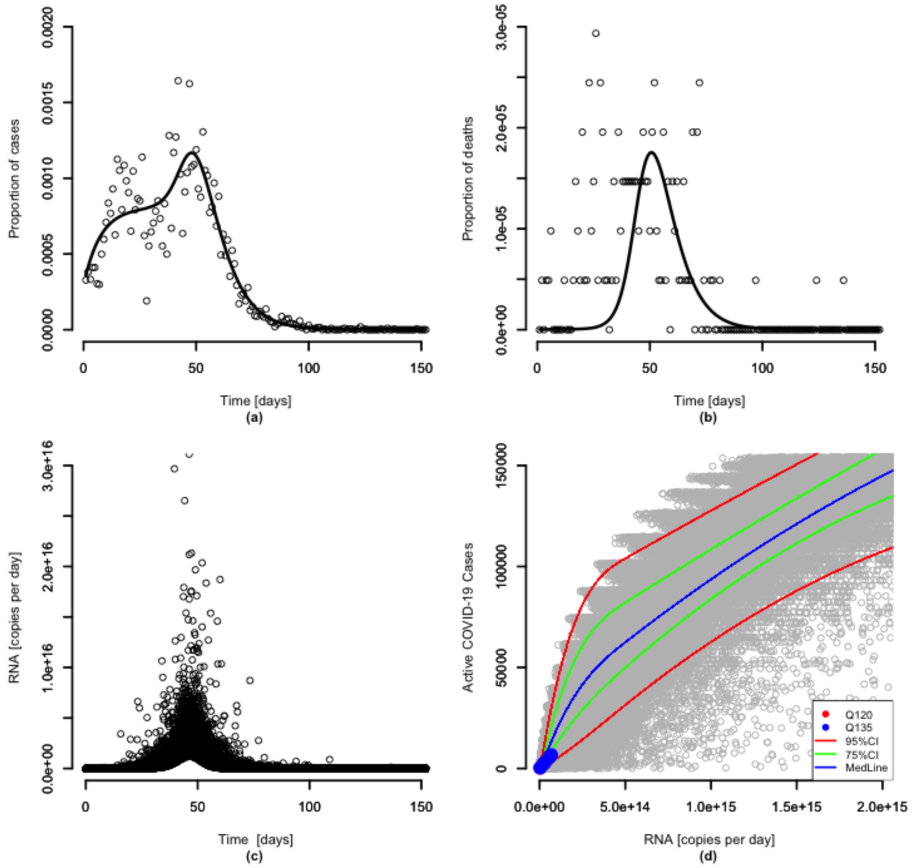

Figure S9F: Considering a viral half-life at an ambient temperature of the drainage of 30 h. Model fit to the proportion of the population that are (a) (left) confirmed positive COVID-19 infections and (b) (right) confirmed deaths from COVID-19 infections for zone 8. (c) SEIPR model (1) prediction for the mass rate of SARS-CoV-2 RNA in wastewater over time via Monte-Carlo simulation represented by black points. (d) Plot of the predicted number of active COVID-19 cases versus SARS-CoV-2 RNA mass rate with individual Monte-Carlo simulations represented by grey points; where 75% CI and 95% CI are denoted by the green and red solid lines respectively. Coloured data points denote the measured RNA mass rates and estimated infectious individuals based on equation (8) as presented in Table 4 respectively for an assumed average per capita wastewater rates of 120 L per person per day (red solid points) and 135 L per person per day (blue solid points) for zone 8.

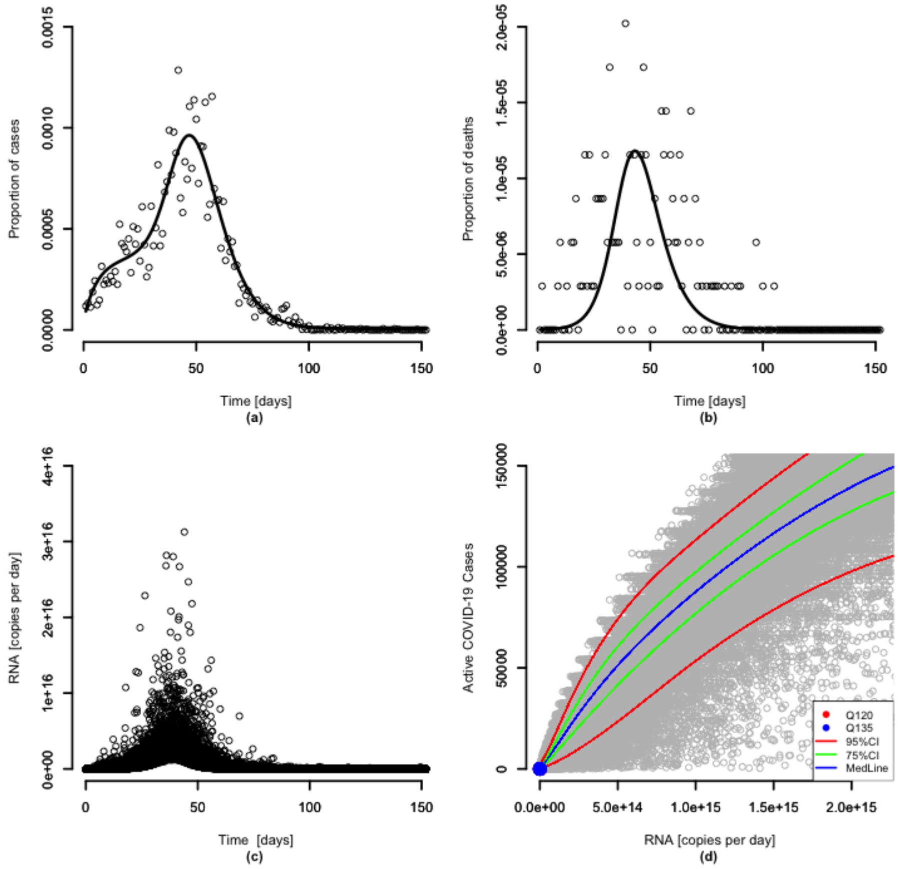

Figure S9G: Considering a viral half-life at an ambient temperature of the drainage of 30 h. Model fit to the proportion of the population that are (a) (left) confirmed positive COVID-19 infections and (b) (right) confirmed deaths from COVID-19 infections for zone 10. (c) SEIPR model (1) prediction for the mass rate of SARS-CoV-2 RNA in wastewater over time via Monte-Carlo simulation represented by black points. (d)Plot of the predicted number of active COVID-19 cases versus SARS-CoV-2 RNA mass rate with individual Monte-Carlo simulations represented by grey points; where 75% CI and 95% CI are denoted by the green and red solid lines respectively. Coloured data points denote the measured RNA mass rates and estimated infectious individuals based on equation (8) as presented in Table 4 respectively for an assumed average per capita wastewater rates of 120 L per person per day (red solid points) and 135 L per person per day (blue solid points) for zone 10.

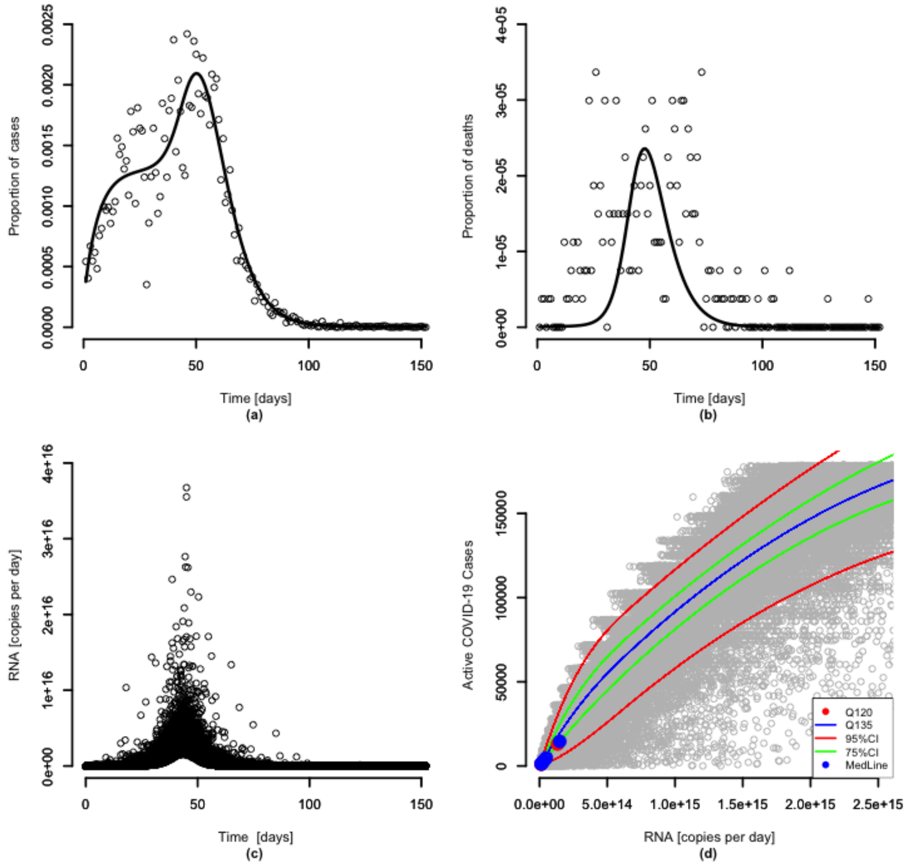

Figure S10A: Considering a viral half-life at an ambient temperature of the drainage of 3 h. Model fit to the proportion of the population that are (a) (left) confirmed positive COVID-19 infections and (b) (right) confirmed deaths from COVID-19 infections for all zones as a single unit. (c) SEIPR model (1) prediction for the mass rate of SARS-CoV-2 RNA in wastewater over time via Monte-Carlo simulation represented by black points. (d) Plot of the predicted number of active COVID-19 cases versus SARS-CoV-2 RNA mass rate with individual Monte-Carlo simulations represented by grey points; where 75% CI and 95% CI are denoted by the green and red solid lines respectively. Coloured data points denote the measured RNA mass rates and estimated infectious individuals based on equation (8) as presented in Table 4 respectively for an assumed average per capita wastewater rates of 120 L per person per day (red solid points) and 135 L per person per day (blue solid points) for all zones as a single unit.

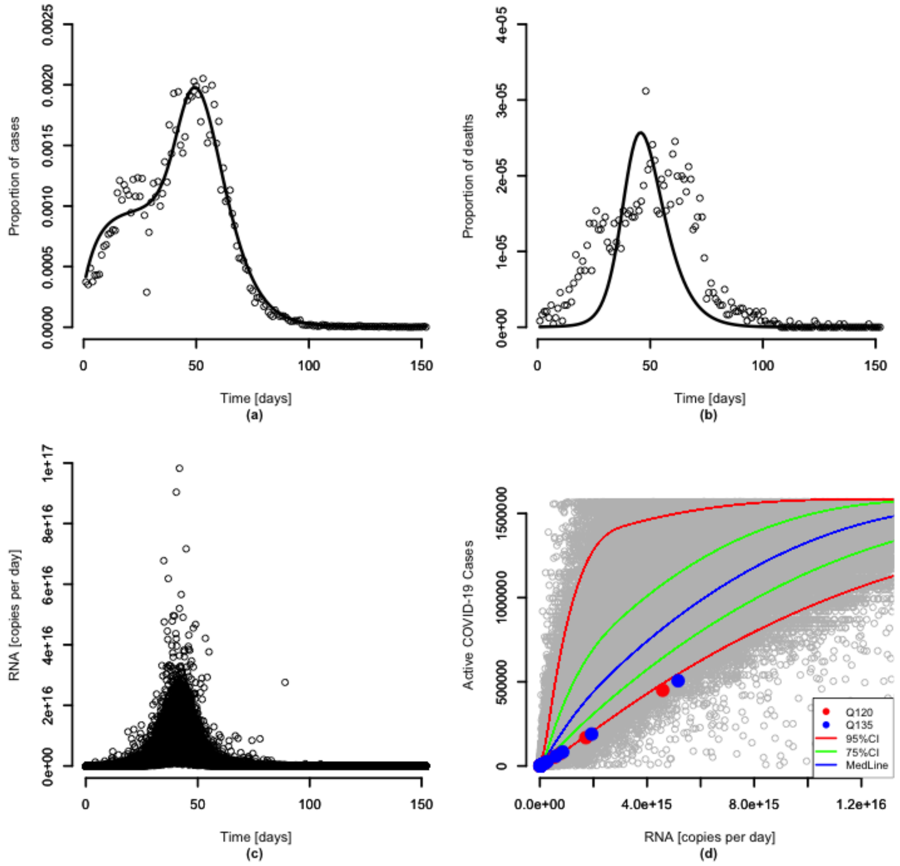

Figure S10B: Considering a viral half-life at an ambient temperature of the drainage of 3 h. Model fit to the proportion of the population that are (a) (left) confirmed positive COVID-19 infections and (b) (right) confirmed deaths from COVID-19 infections for zone 3. (c) SEIPR model (1) prediction for the mass rate of SARS-CoV-2 RNA in wastewater over time via Monte-Carlo simulation represented by black points. (d) Plot of the predicted number of active COVID-19 cases versus SARS-CoV-2 RNA mass rate with individual Monte-Carlo simulations represented by grey points; where 75% CI and 95% CI are denoted by the green and red solid lines respectively. Coloured data points denote the measured RNA mass rates and estimated infectious individuals based on equation (8) as presented in Table 4 respectively for an assumed average per capita wastewater rates of 120 L per person per day (red solid points) and 135 L per person per day (blue solid points) for zone 3.

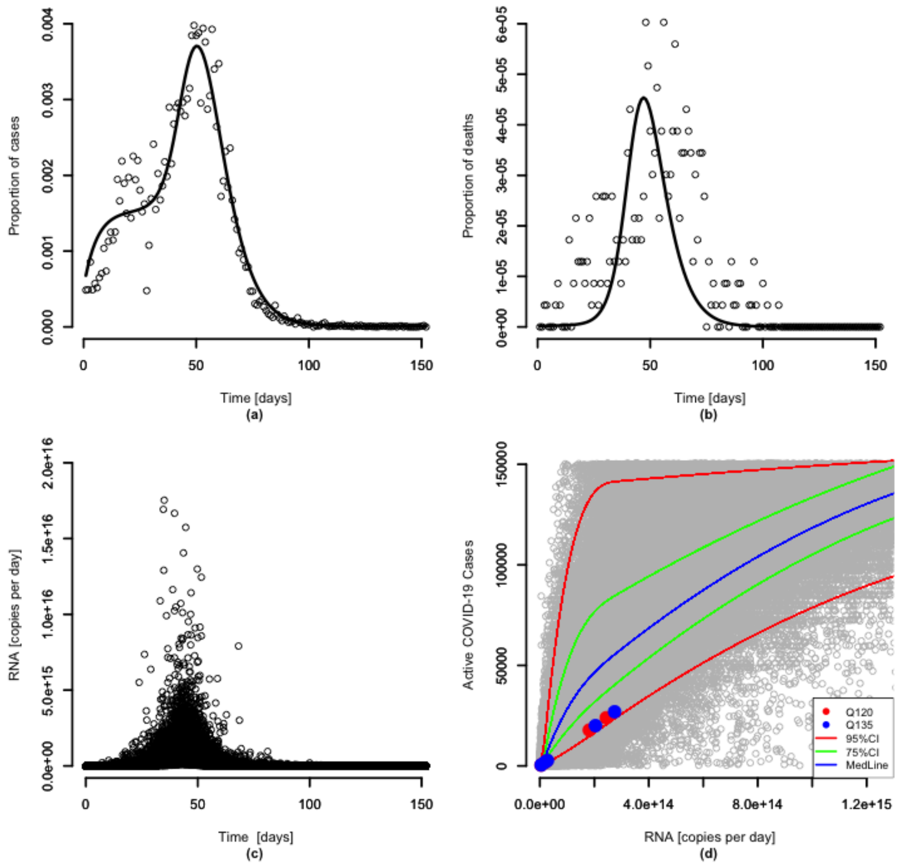

Figure S10C: Considering a viral half-life at an ambient temperature of the drainage of 3 h. Model fit to the proportion of the population that are (a) (left) confirmed positive COVID-19 infections and (b) (right) confirmed deaths from COVID-19 infections for zone 4. (c) SEIPR model (1) prediction for the mass rate of SARS-CoV-2 RNA in wastewater over time via Monte-Carlo simulation represented by black points. (d) Plot of the predicted number of active COVID-19 cases versus SARS-CoV-2 RNA mass rate with individual Monte-Carlo simulations represented by grey points; where 75% CI and 95% CI are denoted by the green and red solid lines respectively. Coloured data points denote the measured RNA mass rates and estimated infectious individuals based on equation (8) as presented in Table 4 respectively for an assumed average per capita wastewater rates of 120 L per person per day (red solid points) and 135 L per person per day (blue solid points) for zone 4.

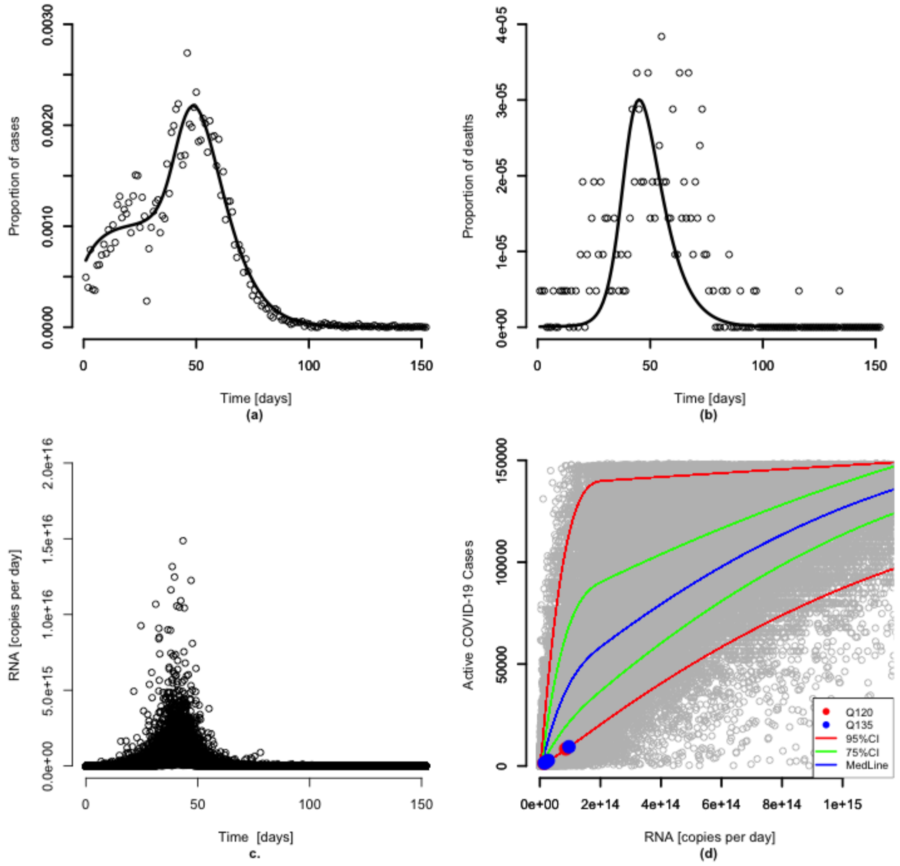

Figure S10D: Considering a viral half-life at an ambient temperature of the drainage of 3 h. Model fit to the proportion of the population that are (a) (left) confirmed positive COVID-19 infections and (b) (right) confirmed deaths from COVID-19 infections for zone 5. (c) SEIPR model (1) prediction for the mass rate of SARS-CoV-2 RNA in wastewater over time via Monte-Carlo simulation represented by black points. (d) Plot of the predicted number of active COVID-19 cases versus SARS-CoV-2 RNA mass rate with individual Monte-Carlo simulations represented by grey points; where 75% CI and 95% CI are denoted by the green and red solid lines respectively. Coloured data points denote the measured RNA mass rates and estimated infectious individuals based on equation (8) as presented in Table 4 respectively for an assumed average per capita wastewater rates of 120 L per person per day (red solid points) and 135 L per person per day (blue solid points) for zone 5.

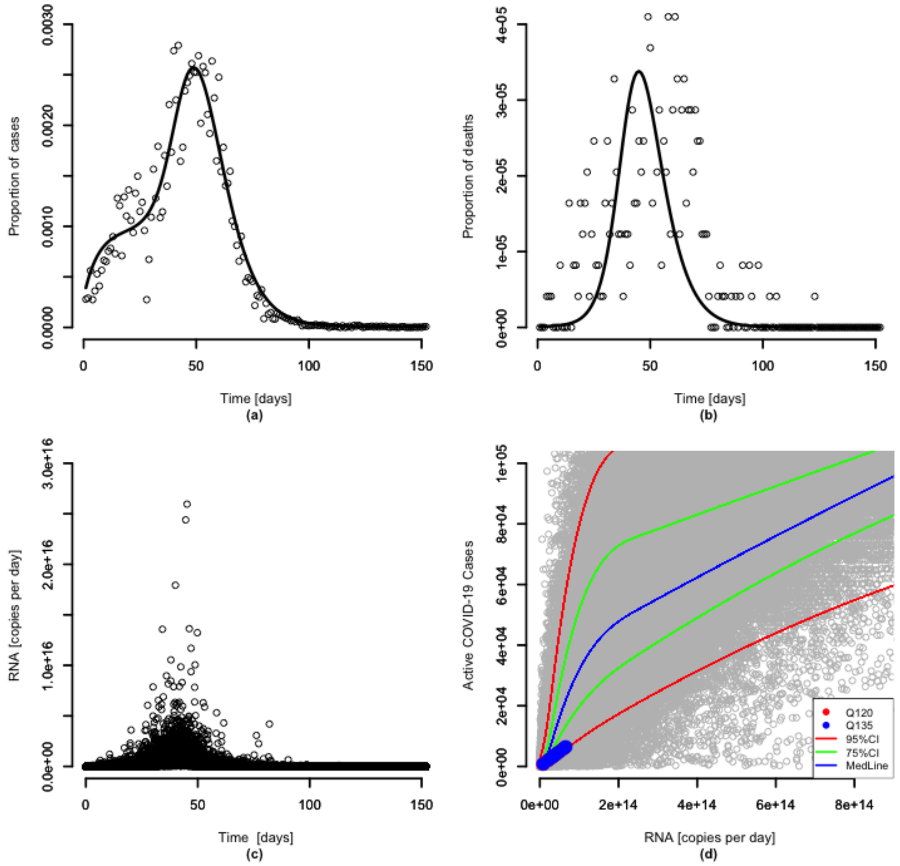

Figure S10E: Considering a viral half-life at an ambient temperature of the drainage of 3 h. Model fit to the proportion of the population that are (a) (left) confirmed positive COVID-19 infections and (b) (right) confirmed deaths from COVID-19 infections for zone 6. (c) SEIPR model (1) prediction for the mass rate of SARS-CoV-2 RNA in wastewater over time via Monte-Carlo simulation represented by black points. (d) Plot of the predicted number of active COVID-19 cases versus SARS-CoV-2 RNA mass rate with individual Monte-Carlo simulations represented by grey points; where 75% CI and 95% CI are denoted by the green and red solid lines respectively. Coloured data points denote the measured RNA mass rates and estimated infectious individuals based on equation (8) as presented in Table 4 respectively for an assumed average per capita wastewater rates of 120 L per person per day (red solid points) and 135 L per person per day (blue solid points) for zone 6.

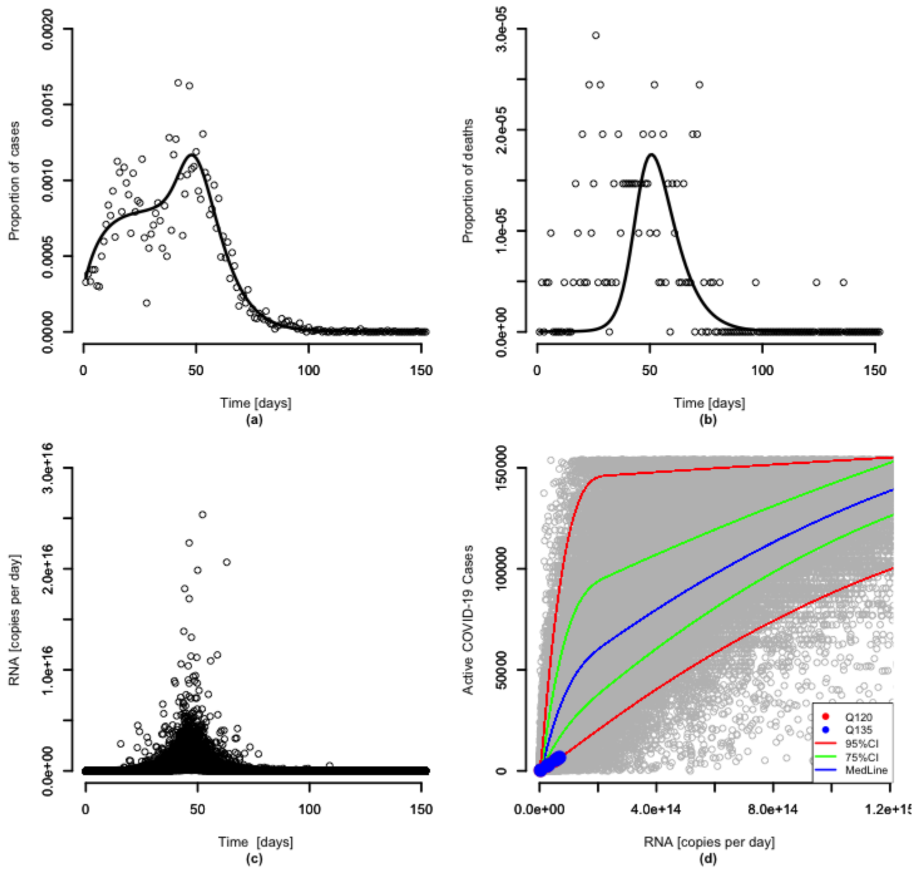

Figure S10F: Considering a viral half-life at an ambient temperature of the drainage of 3 h. Model fit to the proportion of the population that are (a) (left) confirmed positive COVID-19 infections and (b) (right) confirmed deaths from COVID-19 infections for zone 8. (c) SEIPR model (1) prediction for the mass rate of SARS-CoV-2 RNA in wastewater over time via Monte-Carlo simulation represented by black points. (d) Plot of the predicted number of active COVID-19 cases versus SARS-CoV-2 RNA mass rate with individual Monte-Carlo simulations represented by grey points; where 75% CI and 95% CI are denoted by the green and red solid lines respectively. Coloured data points denote the measured RNA mass rates and estimated infectious individuals based on equation (8) as presented in Table 4 respectively for an assumed average per capita wastewater rates of 120 L per person per day (red solid points) and 135 L per person per day (blue solid points) for zone 8.

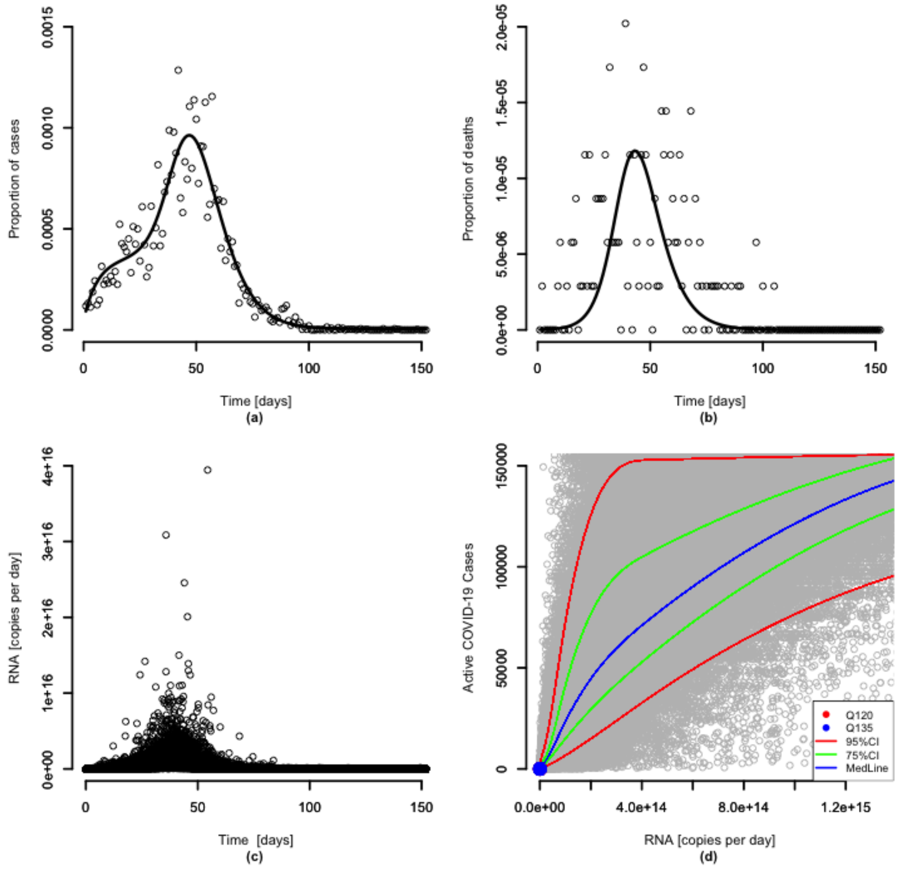

Figure S10G: Considering a viral half-life at an ambient temperature of the drainage of 3 h. Model fit to the proportion of the population that are (a) (left) confirmed positive COVID-19 infections and (b) (right) confirmed deaths from COVID-19 infections for zone 10. (c) SEIPR model (1) prediction for the mass rate of SARS-CoV-2 RNA in wastewater over time via Monte-Carlo simulation represented by black points. (d) Plot of the predicted number of active COVID-19 cases versus SARS-CoV-2 RNA mass rate with individual Monte-Carlo simulations represented by grey points; where 75% CI and 95% CI are denoted by the green and red solid lines respectively. Coloured data points denote the measured RNA mass rates and estimated infectious individuals based on equation (8) as presented in Table 4 respectively for an assumed average per capita wastewater rates of 120 L per person per day (red solid points) and 135 L per person per day (blue solid points) for zone 10.

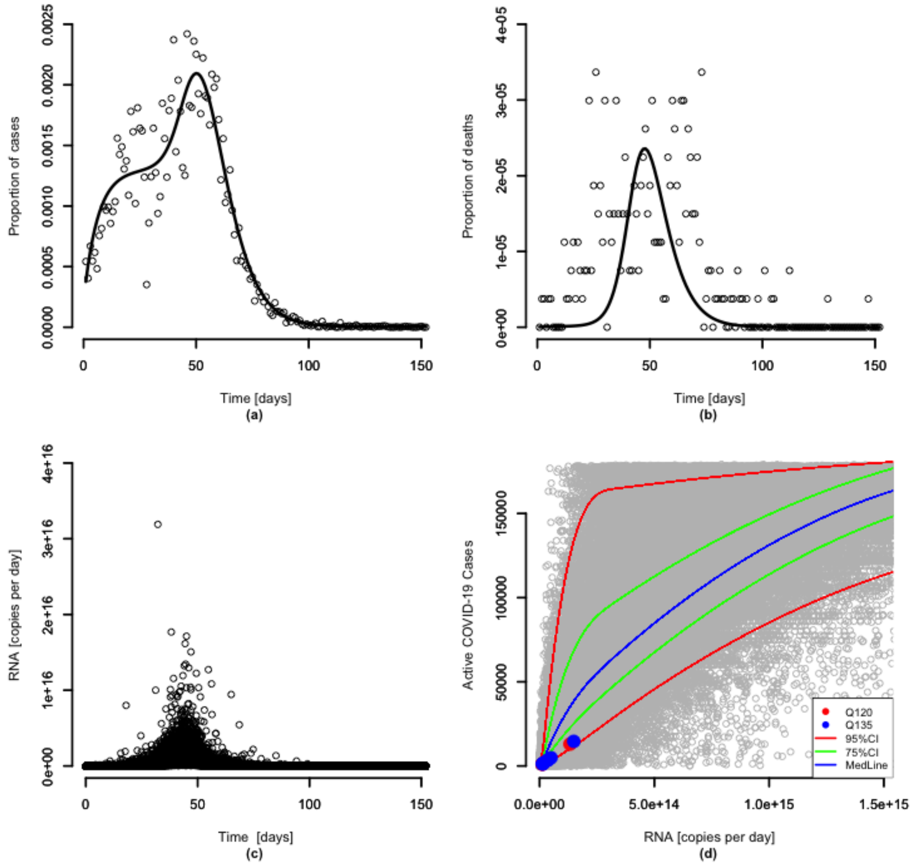

Table S1. Summary of the demographic and environmental characteristics of the urban and rural catchment areas of Nagpur region of India.

Characteristics	Urban $N = 743$	Rural $N = 240$	Significance
Temperature (°C)	29.00 (25.75 - 31.00)	31.00 (29.00 - 33.00)	≤ 0.001
Humidity (%)	38.00 (26.00 - 53.500	32.00 (22.00 - 50.00)	≤ 0.001
Rainfall			
Yes	32 (4.31%)	10 (4.16%)	n.s
No	711 (95.69%)	230 (95.84%)	
Nearby hospital			
Yes	28 (3.77%)	0 (0.00%)	≤ 0.001
No	715 (96.23%)	240 (100%)	
Sanitation			
Good	516 (69.45%)	10 (4.16%)	≤ 0.001
Poor	227 (30.55%)	230 (95.84%)	
Population density			
High	237 (31.90%)	3 (1.25%)	≤ 0.001
Moderation	473 (63.66%)	76 (31.67%)	
Low	33 (4.15%)	161 (67.08%)	
Housing condition			
Very good	585 (78.73%)	0 (0.00%)	≤ 0.001
Good	142 (19.11%)	198 (82.50%)	
Poor	16 (2.15%)	42 (17.50%)	
Drainage system			
Open	38 (5.11%)	240 (100%)	≤ 0.001
Closed	705 (94.89%)	0 (0.00%)	
Livestock domestication			
Yes	131 (17.83%)	289 (99.17%)	≤ 0.001
No	612 (82.37%)	2 (0.83%)	
Market area			
Yes	116 (15.61%)	NA	
No	627 (84.39%)	NA	
Industrial area			
Yes	17 (2.29%)	NA	
No	726 (97.71%)	NA	
Airport*			
Yes	5 (0.67%)	NA	
No	738 (99.33%)	NA	
Rail station*			
Yes	3 (0.40%)	NA	
No	740 (99.60%)	NA	
Bus station*			
Yes	4 (0.54%)	NA	
No	739 (99.46%)	NA	

Data are presented n (%) or median (IQR). NA = not applicable. *: Transportation sites less than 100 meters, a: RT-PCR results for wastewater samples and n.s.: non significance.

Table S2. Summary of the demographic and environmental characteristics of the urban and rural catchment areas of Nagpur region of India.

**Characteristics**	**Zone 1** $N = 98$	**Zone 2** $N = 119$	**Zone 3** $N = 65$	**Zone 4** $N = 47$	**Zone 5** $N = 63$
Rainfall					
Yes	3 (3.06%)	0 (0.00%)	0 (0.00%)	0 (0.00%)	0 (0.00%)
Nearby hospital					
Yes	6 (6.12%)	5 (4.20%)	2 (3.08%)	2 (4.26%)	1 (1.59%)
Sanitation					
Good	95(96.94%)	95 (79.83%)	53 (81.54%)	41 (87.23%)	36 (57.14%)
Population density					
High	2 (2.04%)	33 (27.73%)	12 (18.46%)	21 (44.68%)	13 (20.63%)
Moderation	89 (90.82%)	78 (65.55%)	52 (80.00%)	26 (55.32%)	47 (74.60%)
Low	7 (7.14%)	8 (6.72%)	1 (1.54%)	0 (0.00%)	3 (4.76%)
Housing condition					
Very good	92 (93.88%)	97 (81.51%)	61 (93.85%)	44 (93.62%)	51 (80.95%)
Good	4 (4.08%)	11 (9.24%)	4 (6.15%)	3 (6.38%)	12 (19.05%)
Poor	2 (2.04%)	11 (9.24%)	0 (0.00%)	0 (0.00%)	3 (4.76%)
Drainage system					
Open	88 (89.79%)	97 (81.51%)	65 (100%)	47 (100%)	63 (100%)
Livestock domestication					
Yes	14 (14.28%)	23 (19.33%)	10 (15.38%)	8 (17.02%)	9 (14.29%)
Market area					
Yes	0 (0.00%)	9 (7.56%)	2 (3.08%)	1 (2.13%)	7 (11.11%)
Industrial area					
Yes	1 (1.02%)	8 (6.72%)	1 (1.54%)	1 (2.13%)	0 (0.00%)
Airport*					
Yes	4 (4.08%)	0 (0.00%)	0 (0.00%)	1 (2.13%)	0 (0.00%)
Rail station*					
Yes	0 (0.00%)	0 (0.00%)	0 (0.00%)	0 (0.00%)	0 (0.00%)
Bus station*					
Yes	0 (0.00%)	2 (1.68%)	0 (0.00%)	1 (2.13%)	0 (0.00%)

Data are presented n (%) or median (IQR). NA = not applicable. *: Transportation sites less than 100 meters, a: RT-PCR results for wastewater samples and n.s.: non significance.

Table S3. Summary of the demographic and environmental characteristics of the urban and rural catchment areas of Nagpur region of India.

Characteristics	Zone 6<br>$N = 60$	Zone 7<br>$N = 49$	Zone 8<br>$N = 75$	Zone 9<br>$N = 47$	Zone 10<br>$N = 99$
Rainfall					
Yes	0 (0.00%)	16 (32.65%)	11 (14.67%)	0 (0.00%)	0 (0.00%)
Nearby hospital					
Yes	1 (1.66%)	48 (97.96%)	72 (96.00%)	47 (100%)	7 (7.07%)
Sanitation					
Good	23(38.33%)	24 (48.98%)	33 (44.00%)	32 (68.09.1%)	84 (84.85%)
Population density					
High	58 (96.66%)	29 (61.18%)	27 (36.00%)	29 (61.70%)	13 (13.13%)
Moderation	1(1.66%)	20 (40.82%)	42 (56.00%)	38 (80.85%)	80 (80.80%)
Low	1 (1.66%)	0 (0.00%)	0 (0.00%)	0 (0.00%)	3 (3.03%)
Housing condition					
Very good	51 (85.00%)	25 (51.02%)	47 (62.67%)	29 (61.70%)	88 (88.89%)
Good	9 (15.00%)	24 (48.97%)	28 (37.33%)	39 (82.98%)	8 (8.08%)
Poor	0 (0.00%)	0 (0.00%)	0 (0.00%)	0 (0.00%)	3 (3.03%)
Drainage system					
Open	60 (100%)	49 (100%)	65 (86.67%)	47* (100%)	93 (93.94%)
Livestock domestication					
Yes	20 (33.33%)	10 (20.41%)	19 (25.33%)	11 (23.40%)	7 (7.07%)
Market area					
Yes	0 (0.00%)	9 (18.37%)	2 (2.67%)	1 (2.13%)	7 (7.07%)
Industrial area					
Yes	0 (0.00%)	0 (0.00%)	3 (4.00%)	2 (4.26%)	1 (1.01%)
Airport*					
Yes	0 (0.0%)	0 (0.00%)	0 (0.00%)	0 (0.00%)	0 (0.00%)
Rail station*					
Yes	0 (0.00%)	0 (0.00%)	0 (0.00%)	0 (0.00%)	0 (0.00%)
Bus station*					
Yes	0 (0.00%)	2 (4.08%)	0 (0.00%)	0 (0.00%)	0 (0.00%)

Data are presented n (%) or median (IQR). NA = not applicable. *: Transportation sites less than 100 meters, a: RT-PCR results for wastewater samples and n.s.: non significance.
